# DNA Methylation Age and Physical and Cognitive Aging

**DOI:** 10.1093/gerona/glz246

**Published:** 2019-10-20

**Authors:** Jane Maddock, Juan Castillo-Fernandez, Andrew Wong, Rachel Cooper, Marcus Richards, Ken K Ong, George B Ploubidis, Alissa Goodman, Diana Kuh, Jordana T Bell, Rebecca Hardy

**Affiliations:** 1 MRC Unit for Lifelong Health and Ageing at UCL, Institute of Cardiovascular Science, University College London, UK; 2 CLOSER, UCL Institute of Education, University College London, UK; 3 Department of Twin Research and Genetic Epidemiology, King’s College London, UK; 4 Musculoskeletal Science and Sports Medicine Research Centre, Department of Sport and Exercise Sciences, Faculty of Science and Engineering, Manchester Metropolitan University, UK; 5 MRC Epidemiology Unit and Department of Paediatrics, Wellcome Trust-MRC Institute of Metabolic Science, University of Cambridge School of Clinical Medicine, UK; 6 Centre for Longitudinal Studies, UCL Institute of Education, University College London, UK

**Keywords:** Cognitive aging, Functional performance, Normative aging, Physical performance

## Abstract

**Background:**

DNA methylation (DNAm) age acceleration (AgeAccel) has been shown to be predictive of all-cause mortality but it is unclear what functional aspect(s) of aging it captures. We examine associations between four measures of AgeAccel in adults aged 45–87 years and physical and cognitive performance and their age-related decline.

**Methods:**

AgeAccelHannum, AgeAccelHorvath, AgeAccelPheno, and AgeAccelGrim were calculated in the Medical Research Council National Survey of Health and Development (NSHD), National Child Development Study (NCDS) and TwinsUK. Three measures of physical (grip strength, chair rise speed, and forced expiratory volume in one second [FEV_1_]) and two measures of cognitive (episodic memory and mental speed) performance were assessed.

**Results:**

AgeAccelPheno and AgeAccelGrim, but not AgeAccelHannum and AgeAccelHorvath were related to performance in random effects meta-analyses (*n* = 1,388–1,685). For example, a 1-year increase in AgeAccelPheno or AgeAccelGrim was associated with a 0.01 mL (95% confidence interval [CI]: 0.01, 0.02) or 0.03 mL (95% CI: 0.01, 0.05) lower mean FEV_1_ respectively. In NSHD, AgeAccelPheno and AgeAccelGrim at 53 years were associated with age-related decline in performance between 53 and 69 years as tested by linear mixed models (*p* < .05). In a subset of NSHD participants (*n* = 482), there was little evidence that change in any AgeAccel measure was associated with change in performance conditional on baseline performance.

**Conclusions:**

We found little evidence to support associations between the first generation of DNAm-based biomarkers of aging and age-related physical or cognitive performance in midlife to early old age. However, there was evidence that the second generation biomarkers, AgeAccelPheno and AgeAccelGrim, could act as makers of an individual’s healthspan as proposed.

The worldwide demographic shift towards an aging population is accompanied by an increase in life expectancy; however, the quality of these extra years remains unclear (1–3). Aging is a dynamic and complex process characterized by an array of cellular and molecular changes which accumulate over the life course to manifest as impaired function and an increased susceptibility to multiple chronic diseases and death (4). The heterogeneity in age-related disease and functional capability cannot be explained by chronological age (CA) alone (5). Therefore, a measure of biological age that can capture the aging process beyond what is represented by CA may identify people at risk of functional impairment, providing an insight into their health-related quality of life.

Numerous biomarkers of aging have been proposed including epigenetic biomarkers based on DNA methylation (DNAm) (6–10). Over recent years, a number of DNAm-based biomarkers of aging have been developed (8,11–15). The first generation of these biomarkers were developed to predict CA and include the blood-based Hannum and the multi-tissue Horvath algorithms, which show a high correlation with and small deviation from CA (12,13). More recently, second generation DNAm-based biomarkers of aging have been developed with the specific aim of identifying Cytosine-phosphate-Guanine sites (CpGs) that capture lifespan and healthspan in addition to those displaying changes with chronological time. One of these, the DNAm-based Phenotypic Age (PhenoAge), identified CpGs that predict a composite measure of mortality-related clinical physiological measures and CA (14). Another began by generating surrogate DNAm biomarkers of age-related physiological measures and smoking pack years then regressed time-to-death on these DNAm surrogates and CA to produce the DNAm GrimAge (15).

Having a higher DNAm age independent of CA (denoted age acceleration, AgeAccel), in all of these biomarkers has been shown to be associated with an increased risk of premature all-cause mortality, cardiovascular disease and cancer, with AgeAccelPheno and AgeAccelGrim showing stronger associations than AgeAccelHannum or AgeAccelHorvath (14–21). However, it is unclear what functional aspects of aging these DNAm-based biomarkers of aging capture, and whether they can act as a proxy for an individual’s health beyond mortality and disease.

The maintenance of physical and cognitive performance are vital components of healthy aging and poorer performance has been associated with higher subsequent mortality rates (22,23). Therefore, examining associations between DNAm-based biomarkers of aging and age-related measures of performance and their decline may provide insight into the validity of these as biomarkers of healthy aging. Previous evidence from a small number of studies has been inconsistent and focused on the first generation of DNAm-based biomarkers (24–28). Two studies examining AgeAccelHannum and AgeAccelHorvath and change in physical and cognitive performance were either sex-specific, had small sample sizes and/or did not examine a wide range of performance measures (25,27). Another study examining a range of performance measures among 70-year olds observed cross-sectional but not longitudinal associations between AgeAccelHorvath, AgeAccelHannum and poorer grip strength, lung function and cognitive performance (24).

We used data from the Medical Research Council National Survey of Health and Development (NSHD; 1946 British birth cohort), National Child Development Study (NCDS;1958 British birth cohort) and TwinsUK Registry to examine associations between four DNAm-based biomarkers of aging (AgeAccelHannum, AgeAccelHorvath, AgeAccelPheno, AgeAccelGrim) at ages 45–87 years and a range of physical and cognitive performance measures. We also examine if these DNAm-based biomarkers of aging are associated with decline over 16 years of follow-up in any of the performance measures in NSHD.

## Methods

### Participants

Participants from three cohorts (NSHD, NCDS and TwinsUK) have all been described in detail previously (29–32). Eligible participants had information on DNAm and at least one measure of physical and cognitive performance at the same or a later time point. In NSHD, DNAm and physical and cognitive performance were measured when participants were 53 (*n* = 1,375) and 60–64 years (*n* = 672). Of the participants with DNAm at 53 years, 973 also had physical and cognitive performance measured at 69 years. Participants from NCDS had DNAm and lung function measured at 45 years and cognitive performance measured at 50 years (*n* = 240). For TwinsUK, 120 monozygotic female twins (60 twin pairs) had DNAm profiled when aged 46–87 years and markers of physical performance measured up to 7 years before or after. The mean absolute differences between when DNAm was measured and when grip strength, chair rise speed and lung function was measured was 3, 0.7 and 0.4 years, respectively.

### DNAm-Based Biomarkers of Aging

Blood samples for each cohort were taken as previously described (29,32,33). DNAm was measured at >850,000 CpG sites in each cohort using Infinium MethylationEPIC BeadChips and processed using the ENmix package (34) in R (35) to obtain methylation beta-values. Quality control procedures were applied (Supplementary Material). We estimated four DNAm-based biomarkers of aging in each cohort: DNAm AgeHannum, DNAm AgeHorvath, DNAm PhenoAge, and DNAm GrimAge (12–15). We calculated these four DNAm-based biomarkers of aging using available software (https://labs.genetics.ucla.edu/horvath/dnamage/) with the normalization option and advanced analysis for blood samples. Following the notation of previous publications, CA-independent DNAm-based biomarkers were calculated within the software and denoted as AgeAccelHannum, AgeAccelHorvath, AgeAccelPheno, and AgeAccelGrim, all in units of a year. Estimated blood cell counts (naive and exhausted CD8+ T-lymphocytes, CD4+ T-lymphocytes, B cells, natural killer cells, monocytes, and granulocytes) were also calculated within this software.

### Aging Outcomes

We selected three measures of physical (grip strength, chair rise speed and lung function [forced expiratory volume in one second, FEV_1_]) and two measures of cognitive performance (episodic memory and mental speed), each of which were available in NSHD and at least one other study. All these performance measures were available in NSHD. Cognitive performance and FEV_1_ were measured in NCDS, while TwinsUK collected the three physical performance measures. Details of how each of these measures were assessed is outlined in Supplementary Material.

### Covariates

We selected the covariates a priori based on previous studies (24,27) to include in sensitivity analyses: body mass index (BMI), height (m), smoking status and socioeconomic position indicated by either occupational social class or income. We used covariates measured at the same time as the blood samples. For longitudinal analyses in NSHD, we included time-varying BMI and smoking status measured at 53, 60–64, and 69 years. Details of how each of these covariates were measured is outlined in Supplementary Material.

### Statistical Analyses

All analyses were conducted using the four AgeAccel biomarkers. Further mention of AgeAccel refers to all biomarkers unless specified.

First, we examined associations between AgeAccel and each performance measure within each cohort. Linear regression models were used in NSHD and NCDS and linear mixed models in TwinsUK, including a random effect for twin pair to account for familial effects. We adjusted for sex (in NSHD and NCDS) and CA (in months). We tested for interaction between AgeAccel and sex by including a multiplicative term. We tested for the presence of nonlinear associations by including a quadratic term of AgeAccel in regression models. Results from within-cohort analyses were then combined using random effects meta-analyses. AgeAccel was available in NSHD at two different time points: 53 and 60–64 years. Therefore, we subsequently conducted the primary meta-analyses using data collected at 53 years with a sensitivity analysis including data from 60–64 years.

Second, in NSHD we used linear mixed models with random intercepts and slopes to examine if physical and cognitive performance measures between 53 and 69 years were associated with AgeAccel at 53 years. We included AgeAccel, sex, and CA (in months) at the 53-year measurement as fixed effects. We then tested interactions between time and AgeAccel at 53 years using a log-likelihood ratio test to determine whether it was associated with rate of decline in the performance measures.

Third, in NSHD, we examined if change in AgeAccel between 53 and 60–64 years was associated with change in performance between 53 and 60–64 years. Linear regression models with Δperformance (ie, performance at 60–64 years–performance at 53 years) as the outcome and ΔAgeAccel as the exposure were used. Models were adjusted for performance at 53 years.

Sensitivity analyses were performed repeating each set of analysis with adjustment for BMI, height, smoking status and social class or income. Since the blood-based AgeAccel measures are correlated with cell composition (14,15,17) and cell composition changes with age, we explored cell-intrinsic (17) associations by adjusting for estimated cell counts (naïve and exhausted CD8+ T-lymphocytes, CD4+ T-lymphocytes, B cells, natural killer cells, monocytes, and granulocytes) in sensitivity analyses.

## Results

Table 1 outlines characteristics of the study participants. The correlation coefficients between the different AgeAccel measures were similar within each study. AgeAccelHannum, AgeAccelHorvath, and AgeAccelPheno were moderately correlated (ranging from *r* = .33 for AgeAccelHannum and AgeAccelPheno in TwinsUK to *r* = .58 for AgeAccelHannum and AgeAccelPheno in NCDS). AgeAccelGrim was also moderately correlated with AgeAccelPheno within each study (ranging from *r* = .36 in NSHD 60–64 years to *r* = .39 to *r* = .40 in all other studies). However, AgeAccelGrim had weak correlations with AgeAccelHannum within each study (*r* = ≤.25) and no correlation with AgeAccelHorvath (*r* = ≤.06 in NSHD at 60–64 years, NCDS and TwinsUK *r* = .13 in NSHD at 53 years).

**Table 1.** Characteristics of Study Members by Cohort at Time of DNA Extraction

	NSHD 53 y		NSHD 60–64 y		NCDS		TwinsUK
	Male *n* = 655	Female *n* = 720	Male *n* = 345	Female *n* = 327	Male *n* = 112	Female *n* = 128	Female *n* = 120
Year(s) at DNA extraction	1999		2006–2010		2003		2008–2015
Age (y)	53.4 (0.2)	53.5 (0.2)	63.0 (1.1)	63.1 (1.0)	45.1 (0.36)	45.1 (0.37)	64.6 (9.3)
DNAm AgeHannum (y)	43.1 (4.3)	41.6 (4.0)	53.4 (4.5)	50.4 (3.8)	36.1 (3.2)	35.7 (3.6)	51.8 (8.5)
AgeAccelHannum	0.79 (4.28)	–0.66 (3.95)	1.50 (4.43)	–1.53 (3.69)	0.25 (3.16)	–0.22 (3.59)	–0.06 (3.65)
DNAm AgeHorvath (y)	50.7 (4.2)	49.6 (3.9)	58.9 (4.7)	57.2 (4.1)	45.1 (3.5)	44.0 (3.9)	58.7 (8.1)
AgeAccelHorvath	0.54 (4.15)	–0.54 (3.86)	0.79 (4.65)	–0.83 (4.00)	0.58 (3.47)	–0.51 (3.94)	–0.07 (4.0)
PhenoAge (y)	39.0 (5.6)	38.9 (5.6)	48.9 (5.9)	47.7 (5.8)	37.9 (5.6)	38.6 (5.0)	56.8 (10.8)
AgeAccelPheno	0.05 (5.60)	–0.02 (5.60)	0.57 (5.86)	–0.84 (5.76)	–0.37 (5.60)	0.32 (4.90)	0.47 (5.74)
GrimAge (y)	58.0 (5.14)	55.3 (4.81)	64.6 (4.6)	61.1 (4.3)	48.17 (4.92)	46.70 (4.43)	59.8 (7.8)
AgeAccelGrim	1.40 (5.14)	–1.28 (4.79)	1.53 (4.4)	–1.67 (4.10)	0.82 (4.91)	–0.71 (4.39)	–0.19 (3.02)
Body mass index (kg/m^2^)	27.4 (4.0)	27.3 (5.0)	28.2 (4.2)	28.0 (4.9)	25.2 (3.6)	24.0 (4.3)	26.8 (5.2)
Height (m)	1.75 (0.07)	1.61 (0.06)	1.75 (0.07)	1.62 (0.06)	1.77 (0.07)	1.64 (0.07)	1.62 (0.07)
% current smokers	22.9	25.4	10.5	12.6	19.6	24.2	5.0
% Nonmanual social class/income ≥£25,000*	63.6	69.8	66.9	74.1	70.5	69.5	58.1
Grip strength (kg)	48.3 (12.6)	27.7 (8.3)	45.6 (11.6)	26.4 (7.6)	–	–	24.0 (6.6)
Chair rise speed (stands/min)	32.0 (10.0)	30.0 (9.3)	26.0 (7.0)	24.3 (6.9)	–	–	34.7 (11.0)
FEV_1_	3.26 (0.59)	2.31 (0.45)	3.07 (0.68)	2.18 (0.44)	3.84 (0.68)	2.68 (0.67)	2.28 (0.52)
Number of words recalled	23.2 (6.1)	24.5 (6.2)	23.6 (6.0)	25.6 (5.7)	6.59 (1.52)	6.78 (1.37)	–
Total number of letters scanned	275.2 (73.8)	291.8 (76.0)	258.0 (71.4)	272.2 (69.5)	342.78 (105.5)	348.13 (96.10)	–

*Note*: Values are mean (*SD*) unless stated. AgeAccel = age acceleration.

*Occupational social class for NSHD and NCDS, income for TwinsUK.

### Associations Between AgeAccel and Physical and Cognitive Performance Across the Three Cohorts

**Figure 1.**
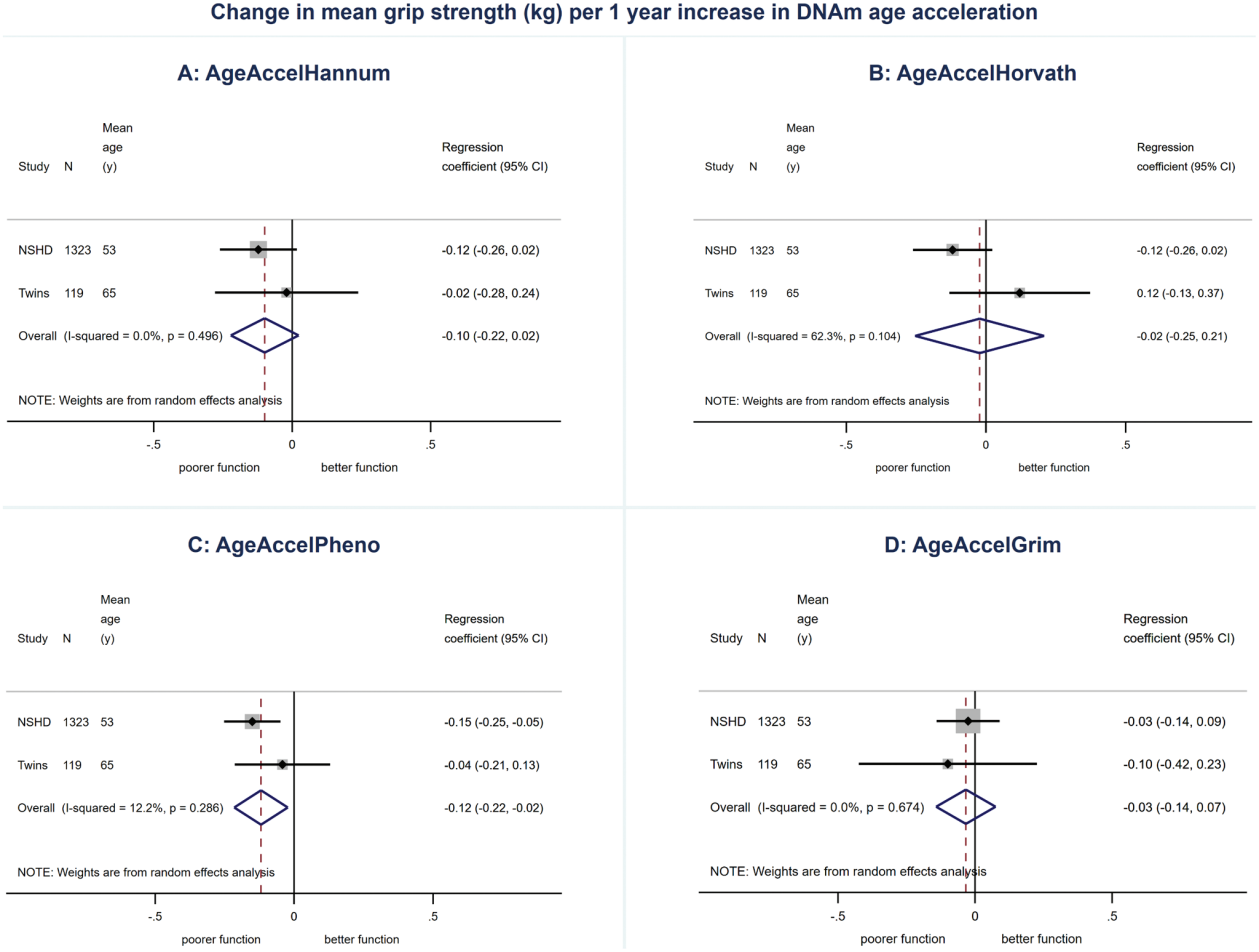
Association between (**A**) AgeAccelHannum, (**B**) AgeAccelHorvath, (**C**) AgeAccelPheno, (**D**) AgeAccelGrim and grip strength adjusted for sex and age.

**Figure 2.**
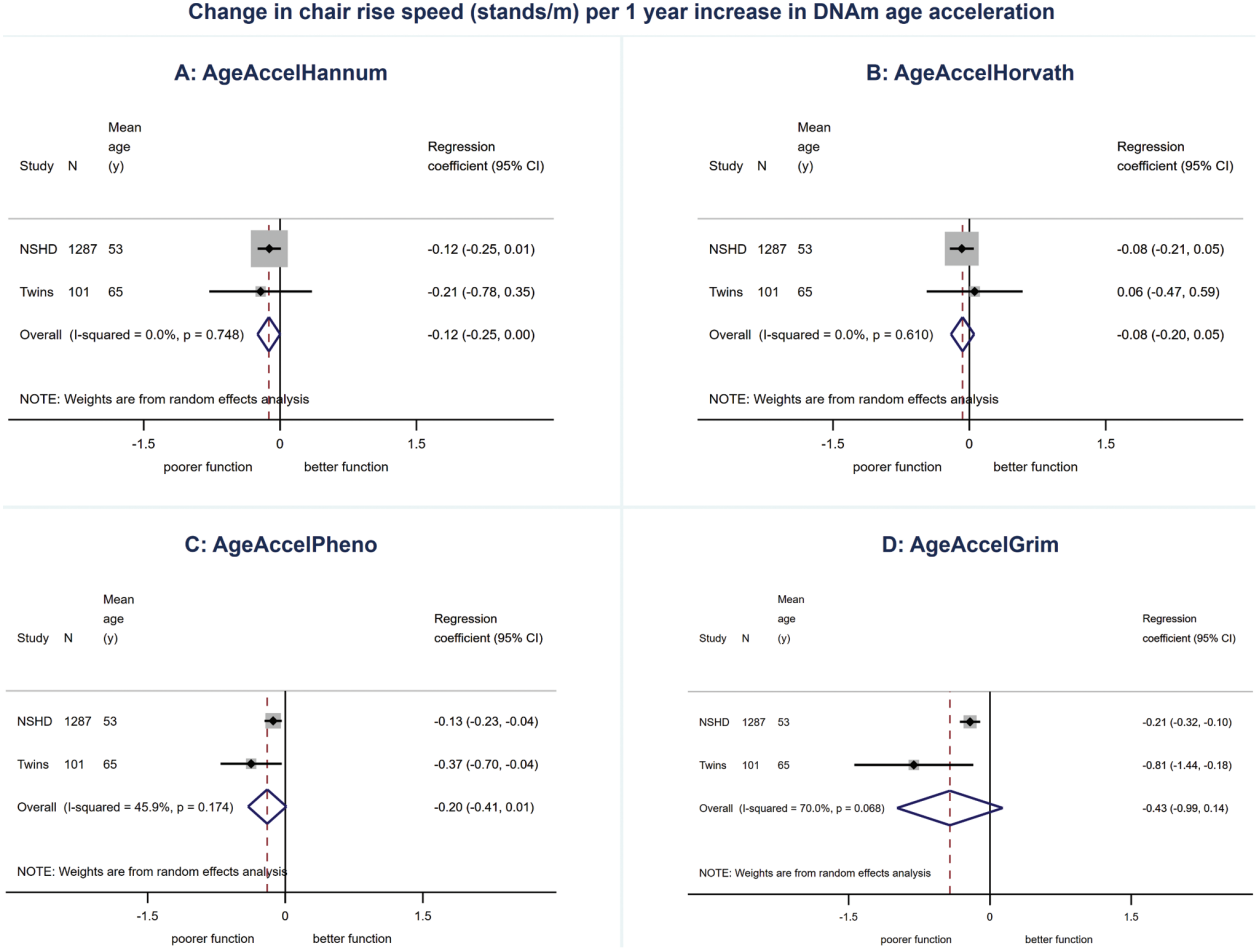
Association between (**A**) AgeAccelHannum, (**B**) AgeAccelHorvath, (**C**) AgeAccelPheno, (**D**) AgeAccelGrim and chair rise speed adjusted for sex and age.

**Figure 3.**
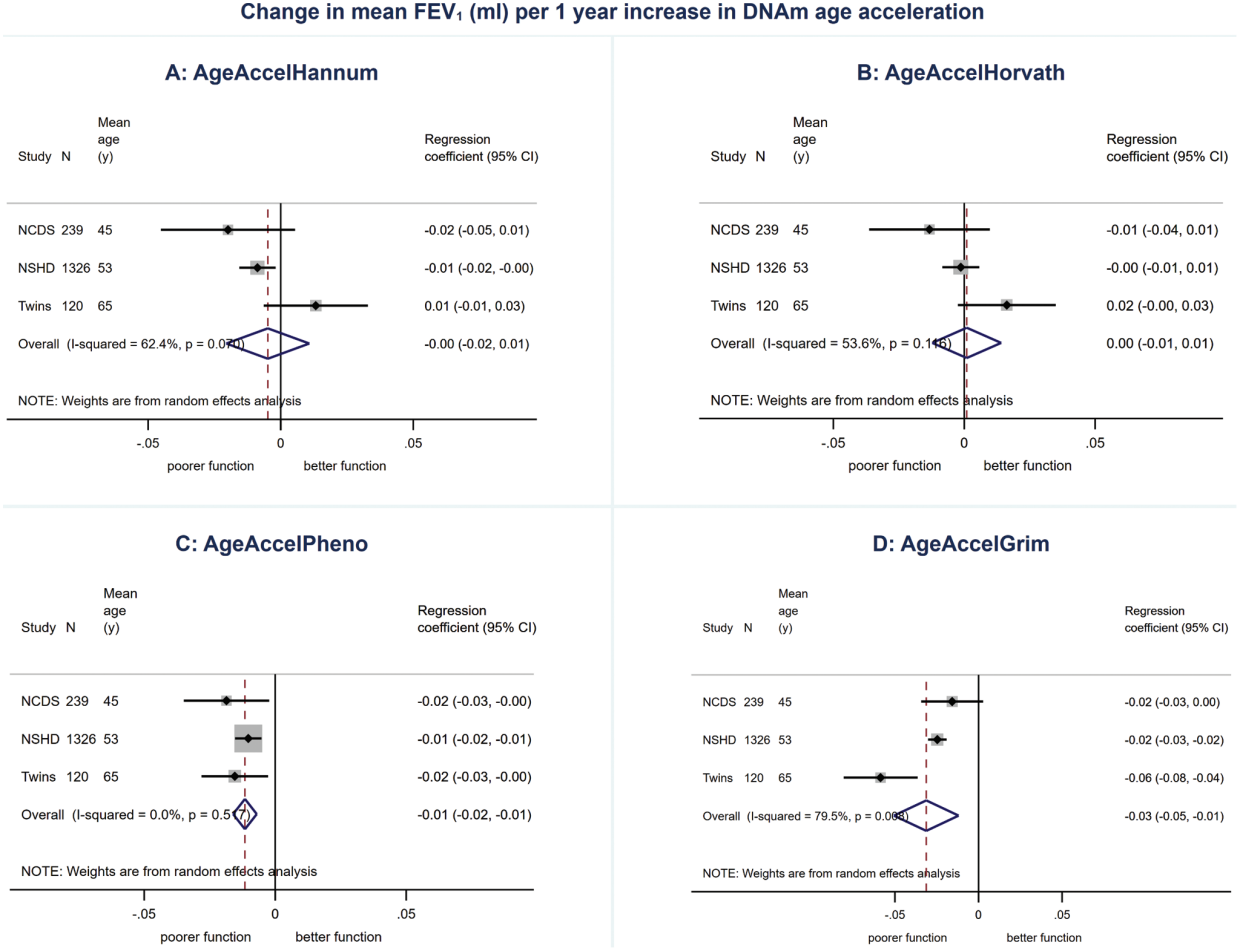
Association between (**A**) AgeAccelHannum, (**B**) AgeAccelHorvath, (**C**) AgeAccelPheno, (**D**) AgeAccelGrim and FEV_1_ adjusted for sex and age.

**Figure 4.**
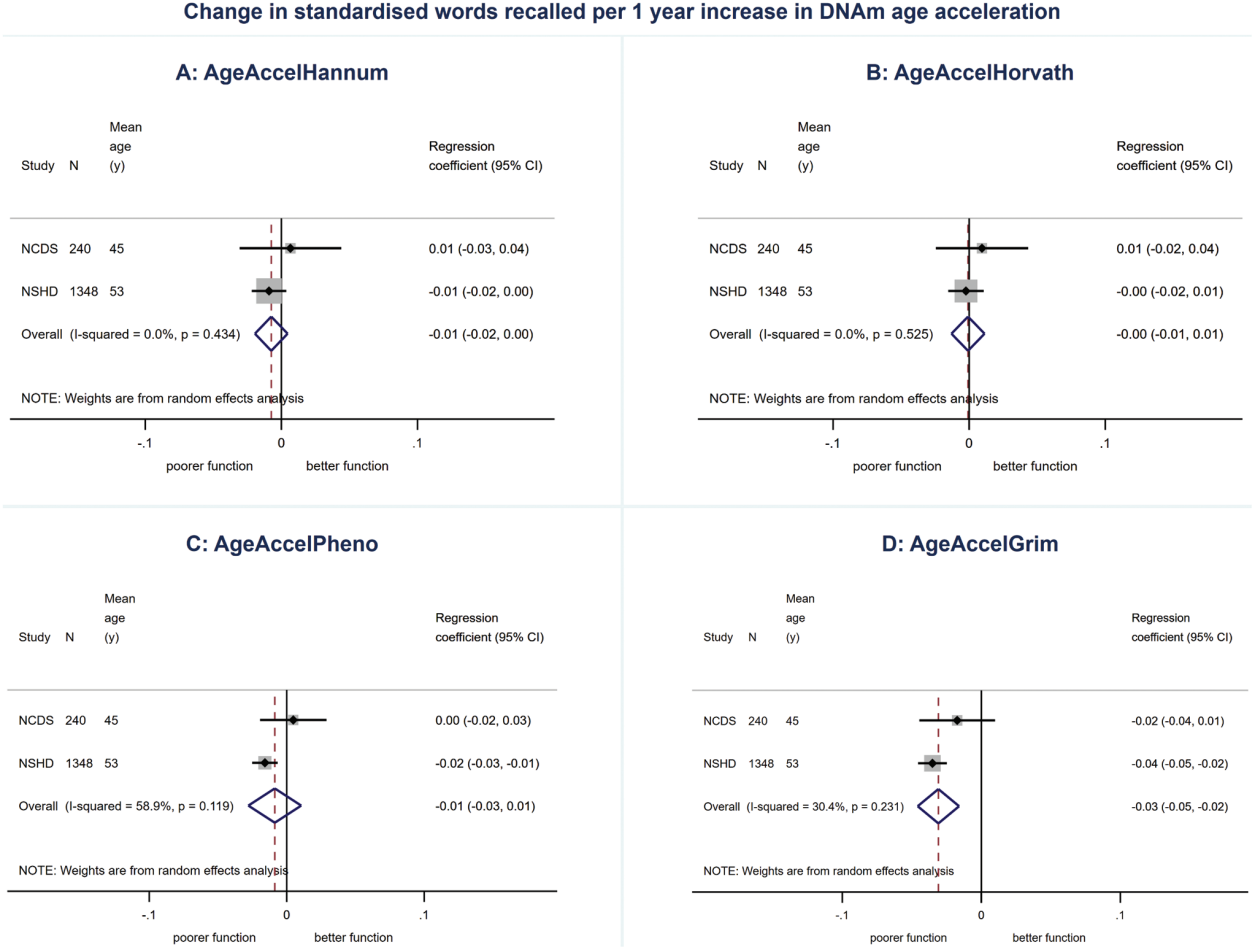
Association between (**A**) AgeAccelHannum, (**B**) AgeAccelHorvath, (**C**) AgeAccelPheno, (**D**) AgeAccelGrim and standardized total number of words recalled adjusted for sex and age.

**Figure 5.**
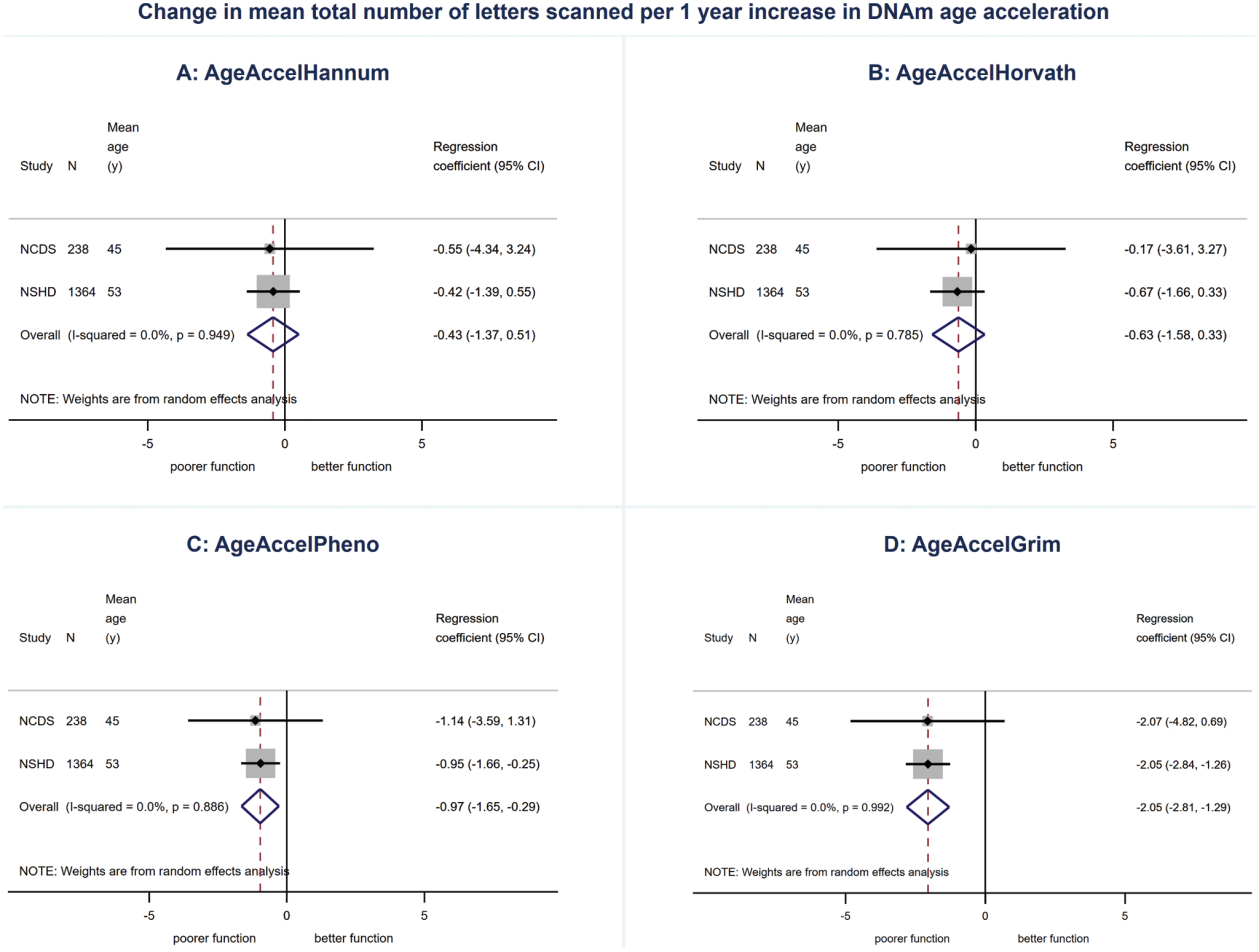
Association between (**A**) AgeAccelHannum, (**B**) AgeAccelHorvath, (**C**) AgeAccelPheno, (**D**) AgeAccelGrim and total number of letters scanned adjusted for sex and age.

We found no evidence for associations between AgeAccelHannum or AgeAccelHorvath and physical or cognitive performance in meta-analyses (Figures 1–5, Supplementary Tables 1 and 2). There was evidence of a nonlinear association (*p*_linearity_ = .04) and sex interaction (*p*_interaction_ = .01) between AgeAccelHorvath and FEV_1_. When stratified by sex, effect sizes for the association between AgeAccelHorvath and FEV_1_ were in opposite directions. In women, higher AgeAccelHorvath was associated with lower FEV_1_, but in men with better FEV_1_ (Supplementary Table 2).

Higher AgeAccelPheno was associated with weaker grip strength, lower FEV_1_ and slower mental speed (Supplementary Table 3 and Figures 1–5). Higher AgeAccelGrim was associated with lower FEV_1_, poorer episodic memory and slower mental speed (Supplementary Table 4 and Figures 1–5). There was no evidence for a sex interaction with AgeAccelPheno or AgeAccelGrim and any of the performance measures. Estimates for the association between AgeAccelGrim and physical and cognitive performance tended to be stronger than AgeAccelPheno (Supplementary Tables 3 and 4 and Figures 1–5). For example, a 1-year increase in AgeAccelPheno was associated with –0.97 (95% confidence interval [CI]: –1.65 to –0.29) mean reduction in number of letters scanned while AgeAccelGrim with a –2.05 (95% CI: –2.81 to –1.29) reduction.

Adjustment for covariates attenuated the association between AgeAccelPheno and grip strength but did not affect the overall conclusions for other associations (Supplementary Figures 6–10). Adjusting for cell composition did not affect the majority of the results; however, the association between AgeAccelGrim and chair rise speed became stronger due to the study estimates becoming less heterogeneous (Supplementary Figures 1–5).

There were no major differences in conclusions for AgeAccelHannum and AgeAccelHorvath between meta-analyses including NSHD participants at 53 years and the ones including participants at 60–64 years with only minor differences in estimates and evidence for a nonlinear association between AgeAccelHannum and mental speed (*p*_linearity_ = .04, Supplementary Table 1) and no evidence for nonlinearity or sex interactions between AgeAccelHorvath and FEV_1_ (Supplementary Table 2). Overall, estimates for the associations between AgeAccelPheno and performance measures were weaker when data on NSHD participants from age 60–64 years were compared with data from 53 years in meta-analyses. This was due to the weaker associations observed at 60–64 years compared with 53 years. Conversely, estimates for the associations between AgeAccelGrim and performance measures were stronger when including NSHD participants at 60–64 years compared with 53 years.

### Longitudinal Associations Between AgeAccel at 53 Years and Change in Physical and Cognitive Performance Between 53 and 69 Years in NSHD

There was an overall decline in mean physical and cognitive performance between 53 and 69 years in NSHD (Supplementary Table 5). There was no evidence that AgeAccelHorvath at 53 years was related to any physical or cognitive performance measure between 53 and 69 years (Table 2). Higher AgeAccelHannum at 53 years was associated with poorer physical performance and episodic memory over the 16-year period, but not with the rate of decline (*p*_interaction_ ≥ .17). Higher AgeAccelPheno and AgeAccelGrim at 53 years were also associated with a poorer chair rise speed and cognitive performance measures at 53 and 69 years (Table 2), but not with decline. For grip strength, higher AgeAccelPheno at 53 years was related to poorer performance at all ages, while higher AgeAccelGrim was not associated with performance at 53 years but was associated with greater decline between 53 and 69 years. Thus, by age 69 years, a 1-year higher AgeAccelGrim was associated with a –0.25 kg (95% CI: –0.37 to –0.14) weaker grip strength. There was evidence that higher AgeAccelPheno and AgeAccelGrim at 53 years were associated with lower FEV_1_ at 53 years and with a faster decline where the negative association slightly strengthened over time. By 69 years, a 1-year higher AgeAccelPheno at 53 years was associated with a –0.016 mL (95% CI: −0.021 to −0.010) lower mean FEV_1_ compared with a –0.010 mL (95% CI: −0.015 to −0.005) at 53 years. Similarly, for AgeAccelGrim at 53 years, the association was –0.02 mL (95% CI: −0.03 to −0.02) at 69 years compared with –0.04 (95% CI: −0.05 to −0.04) at 53 years. Adjusting for cell composition did not affect the results for AgeAccelHorvath, AgeAccelPheno, or AgeAccelGrim but did attenuate the results for AgeAccelHannum (Supplementary Table 6). Adjustment for covariates attenuated the associations but did not change the overall conclusions for AgeAccelPheno or AgeAccelGrim (Supplementary Table 7).

**Table 2.** Association Between DNAm Age Acceleration at 53 Years and Longitudinal Change in Performance (53–69 years) in NSHD

	Grip strength (kg) (*n* = 1362)		Chair rise speed (stands/min) (*n* = 1334)		FEV_1_ (mL) (*n* = 1359)		Number of words (*n* = 1358)		Total number of letters scanned (*n* = 1368)	
	Estimate (95% CI)	*p*-value	Estimate (95% CI)	*p*-value	Estimate (95% CI)	*p*-value	Estimate (95% CI)	*p*-value	Estimate (95% CI)	*p*-value
AgeAccelHannum	–0.12 (–0.22, –0.02)	.03	–0.101 (–0.197, –0.005)	.04	–0.009 (–0.015, –0.002)	.01	–0.074 (–0.146, –0.001)	.04	–0.55 (–1.38, 0.29)	.20
AgeAccelHorvath	–0.06 (–0.17, 0.04)	.27	–0.05 (–0.18, 0.08)	.49	0.0003 (–0.0065, 0.0071)	.94	–0.02 (–0.09, 0.06)	.68	–0.34 (–1.20, 0.52)	.44
AgeAccelPheno	–0.14 (–0.22, –0.07)	<.001	–010 (–0.17, –0.03)	.01	–0.010* (–0.015, –0.005)	<.001	–0.11 (–0.16, –0.06)	<.001	–1.15 (–1.76, –0.54)	<.001
AgeAccelPheno × time					–0.003** (–0.001, <–0.000)	.04				
AgeAccelGrim	0.004* (–0.110, 0.118)	.92	–0.23 (–0.32, –0.15)	<.001	–0.024* (–0.029, –0.018)	<.001	–0.24 (–0.29, –0.18)	<.001	–2.17 (–2.87, –1.48)	<.001
AgeAccelGrim × time	–0.016** (–0.025, –0.008)	<.001			–0.0010** (–0.0014, –0.0007)	<.001				

Note: All models adjusted for sex and age at 53 years.

Estimates represent difference in outcome which is constant between 53 and 69 years for a 1-year increase in AgeAccel at 53 years unless *p*-value from log-likelihood ratio test comparing models fit with an interaction term for time to models without the interaction term = ≤0.05 then:

*Estimates represent the average difference in outcome at 53 years (i.e. at the intercept) for a 1-year increase in AgeAccel at 53 years.

**Estimates represent the average difference in the linear slope (per year time) between 53 and 69 years for a 1-year increase in AgeAccel at 53 years.

### Associations Between Change in AgeAccel and Change in Physical and Cognitive Performance Between 53 and 60–64 Years in NSHD

In NSHD, 482 participants had AgeAccel information at both 53 and 60–64 years. There was some evidence for an association between greater change in ΔAgeAccelPheno and change in chair rise speed (Table 3), however this was attenuated after accounting for cell composition and additional covariates (Supplementary Tables 8 and 9). There was no association between any of the other ΔAgeAccel measures and Δphysical/cognitive performance between the ages of 53 and 60–64 years (Table 3). However, adjusting for cell composition strengthened associations between ΔAgeAccelGrim and Δgrip strength and Δchair rise speed (Supplementary Table 8).

**Table 3.** Association Between Change in DNAm Age Acceleration and Change in Age-Related Performance Between 53 years and 60–64 years Conditional on Baseline Performance at 53 years

	ΔGrip strength (kg) (*n* = 435)		ΔChair rise (stands/min) (*n* = 418)		ΔFEV_1_ (*n* = 451)		ΔNumber of words recalled (*n* = 456)		ΔTotal number of letters scanned (*n* = 464)	
	Estimate (95% CI)	*p*-value	Estimate (95% CI)	*p*-value	Estimate (95% CI)	*p*-value	Estimate (95% CI)	*p*-value	Estimate (95% CI)	*p*-value
ΔAgeAccelHannum	0.19 (–0.07, 0.44)	.15	0.03 (–0.14, 0.20)	.74	0.006 (–0.007, 0.019)	.34	–0.01 (–0.13, 0.11)	.88	0.49 (–1.19, 2.17)	.56
ΔAgeAccelHorvath	–0.06 (–0.28, 0.16)	.59	–0.12 (–0.27, 0.03)	.12	–0.002 (–0.013, 0.008)	.66	0.06 (–0.05, 0.17)	.25	1.31 (–0.17, 2.79)	.08
ΔAgeAccelPheno	0.03 (–0.14, 0.20)	.72	–0.12 (–0.24, –0.01)	.04	0.006 (–0.003, 0.015)	.17	0.05 (–0.04, 0.13)	.25	0.16 (–0.98, 1.31)	.78
ΔAgeAccelGrim	–0.04 (–0.18, 0.11)	.60	–0.06 (–0.16, 0.04)	.23	0.006 (–0.001, 0.013)	.09	0.04 (–0.03, 0.11)	.27	–0.91 (–1.87, 0.05)	.06

## Discussion

We found evidence of relationships between the second generation of DNAm-based biomarkers of aging (AgeAccelPheno and AgeAccelGrim) and physical and cognitive performance among participants aged 45–87 years that were not observed for the first generation biomarkers (AgeAccelHannum and AgeAccelHorvath). In addition, associations between AgeAccelGrim and subsequent decline in performance was observed.

Identifying a reliable and valid biomarker of aging has the potential to progress the understanding of, and slow the rate of aging (36). While AgeAccel has been heralded as a promising aging biomarker (6), evidence to date has focused on the first generation of DNAm-based biomarkers and has been sparse and inconsistent. Findings from a previous cross-sectional study examining associations between AgeAccelHannum or AgeAccelHorvath in 486 monozygotic twins aged 55–79 years and general cognitive function were in line with our null results (25). In contrast to our findings, a separate study of middle-aged female monozygotic twins (*n* = 24 twin pairs) found some evidence for a cross-sectional association between higher AgeAccelHorvath and lower grip strength (26).

Our results for the first generation of DNAm-based biomarkers of aging should also be compared with previous data from the 1936 Lothian birth cohort (24). Marioni and coworkers observed associations between higher AgeAccelHorvath and poorer physical and cognitive performance at 70 years. Similar to our findings, they found no association between AgeAccelHorvath and decline in performance between 70 and 76 years. Although we did not find any associations for the first generation of DNAm-based biomarkers of aging in our meta-analyses, the direction of the effects are consistent (except for FEV_1_ where we observed an estimate of 0.001 [95% CI: –0.013, 0.011]) with data from the Lothian birth cohort. We included a larger sample size than the Lothian birth cohort (*n* = 1,388*–*1685 vs *n* = ~920); however, most of our participants were younger and it is possible that the cross-sectional association may get stronger with increasing age. Another study examined DNAm AgeHannum and DNAm AgeHorvath in a younger cohort (*n* = 818) (28). The authors observed modest associations between DNAm AgeHannum at 38 years and measures of cognitive function but not grip strength or for DNAm AgeHorvath and any marker of performance (28). The authors found no evidence of an association between change in DNAm AgeHannum between the ages of 26 and 38 years and measures of cognitive or physical performance at 39 years, although change in DNAm AgeHorvath was weakly correlated with cognitive performance (*r* = .11).

One previous study examined the association between AgeAccelHorvath and physical performance using data from a smaller subsample of NSHD women (27). In this study, no associations between AgeAccelHorvath measured using blood samples at 53 years and grip strength or chair rise time at 53 or 60–64 years were observed; unlike results from our study, AgeAccelHorvath at baseline (53 years) was modestly associated with a greater decline in grip strength between 53 and 60–64 years. The 152 women in the previous paper were not included in our study because DNAm was measured using the Infinium Methylation450k BeadChips while our study used Infinium MethylationEPIC BeadChips. In addition, half of these women were selected because they developed breast cancer and thus findings in this subsample may not be representative of all women.

The second-generation DNAm-based biomarkers of aging have been developed more recently with the specific aim of acting as a biomarker for healthspan (AgeAccelPheno) and lifespan (AgeAccelGrim). While AgeAccelPheno and AgeAccelGrim have been associated with age-related disease, and mortality (14,15), to the best of our knowledge, no study to date has examined their associations with physical of cognitive performance. Although all correlations were in the expected direction, it is unclear why we observed associations between AgeAccelPheno and AgeAccelGrim for some but not all age-related performance measures. Over 95% of the participants included in our meta-analyses are aged 65 or younger. It is possible that the variation in both age-related performance and AgeAccel in this age group is small; however, decline in age-related performance has been shown to be evident prior to 65 years (37–41). Furthermore, older participants tend to have a lower AgeAccel than younger participants, indicating the presence of survivor bias (42,43), which would be less evident in our younger sample. In sensitivity analysis for our meta-analysis, including data from NSHD participants at 60–64 years rather than 53 years, weakened the associations between AgeAccelPheno and performance measures because cross-sectional associations were weaker at the older age in NSHD.

Both AgeAccelPheno and AgeAccelGrim, as well as being associated cross-sectionally with performance measures were also associated with decline in FEV_1_ over a 16-year period. Lung function has been shown to decline in NSHD from as early as 43 years (38), which might explain why we observe such a relationship in FEV_1_ but not other performance measures in our relatively younger participants. In addition, AgeAccelGrim at 53 years, although not associated with weaker grip strength cross-sectionally, was associated with grip strength by 69 years. This suggests that even if DNAm-based biomarkers of aging in early midlife do not reflect all current performance measures, they may be related to subsequent decline.

The second generation healthspan and lifespan biomarkers use differences in physiological status among individuals of the same CA to construct the score, unlike the first generation which use CA only. In line with findings for age-related disease (15), we observed the strongest associations for age-related performance for AgeAccelGrim. DNAm AgeHannum is based on 71 CpGs while DNAm AgeHorvath with 353, DNAm PhenoAge with 513 CpGs, and DNAm GrimAge with 1030 CpGs. Of the 513 CpGs for DNAm PhenoAge, 41 overlapped with DNAm AgeHorvath and with 6 DNAm AgeHannum. Furthermore, DNAm AgeHorvath was the only one of the biomarkers that was based on multiple-tissues, with the others using whole blood. Since both blood cell composition and DNAm changes with age (17,44), the blood-based DNAm-based biomarkers of age also reflect age-related changes in cell-type composition (14,15). A previous study found that measures of AgeAccelHorvath and AgeAccelHannum that incorporate blood cell counts give stronger associations with all-cause mortality compared with measures independent of blood cell counts (17). Adjusting for cell composition in our study generally weakened associations, but did not affect the overall conclusions except for associations between AgeAccelHannum and performance in NSHD. In line with previous findings that AgeAccelHannum measures that incorporate blood cell counts outperform measures that exclude them for mortality prediction, our observed associations were considerably attenuated.

To the best of our knowledge, this is the largest study of AgeAccel and physical and cognitive performance to date and the only one that has included the second generation of DNAm-based biomarkers of healthspan and lifespan. The main strengths of our study were the inclusion of participants from three British cohorts, the examination of a range of objective measures of physical and cognitive performance, and the longitudinal performance measures available from three time points in NSHD. However, the sample size remains relatively small and we may still lack power to detect small associations. Despite having three time points for performance measures for longitudinal analyses, this was only possible in NSHD and therefore may lack power. Furthermore, when assessing change in AgeAccel and change in performance over the same 10-year period in NSHD, only two time points were available which adds additional measurement error.

Although participants across the three cohorts are generally representative of the white British population (29–32), selection bias for this specific cross-cohort study may have influenced the observed associations. Participants from these cohorts were selected if they had information on DNAm, outcomes of interest and a range of other health- and age-related variables. If having a lower AgeAccel and higher age-related performance was associated with participation, this could have introduced collider bias where estimates may be positively biased (45). Finally, the performance measures were assessed slightly differently between the cohorts potentially introducing some heterogeneity, although there was little variation in observed effect sizes between the cohorts in most cases. For the meta-analyses, all associations in NSHD were cross-sectional but in NCDS cognitive performance were measured 5 years after DNAm age and performance was measured up to 7 years before or after DNAm age in TwinsUK. It is possible that physical and cognitive performance changed during this period introducing some error in the observed effect sizes. In conclusion, our study found evidence to support the second generation of DNAm-based biomarkers of healthspan and lifespan as a proxy for age-related physical or cognitive performance in mid- to early old age, particularly for lung function. However, these findings should be replicated and current validated measures of physical and cognitive performance should not be replaced by these DNAm-based biomarkers.

## Supplementary Material

glz246_suppl_Supplementary_Material
