# Supplementary material for: DNA Methylation Age and Physical and Cognitive Aging
Source: J Gerontol A Biol Sci Med Sci. 2019 Oct 20;75(3):504–11. doi: 10.1093/gerona/glz246 (PMC8414926; doi:10.1093/gerona/glz246)
Supplement: glz246_suppl_Supplementary_Material [file GERONA_75_3_504_s6.pdf]

DNA methylation age and physical and cognitive ageing
Supplementary materials

DNA methylation quality control procedure

Pre-processing of the DNA methylation arrays were performed in each study using the ssNoob method[1]. In addition, signals with a detection p-value > 1x10-6 and a number of beads <3 were set to missing. Samples with missing data in >5% of the CpGs were excluded. CpGs with missing data in >5% of the samples were excluded. Samples identified with outlier values (more than three standard deviations from the mean or three interquartile ranges below the first or above the third quartiles) in bisulfite intensity, total intensity, or beta-value distribution were excluded. Sample identity was verified estimating the correlation between the 59 SNPs included in the methylation beadchips and imputed genotyped data. After quality control 850 964, 865 128 and 864 858 probes for 2049, 240 and 120 samples remained in NSHD, NCDS and TwinsUK respectively.

Ageing outcomes

Physical performance

Grip strength: In NSHD, grip strength was assessed by trained research nurses using standardised protocols. Participants who were unable to perform the test for health reasons were recorded; however they were excluded from our analyses as the other cohorts did not have this information. A Nottingham electronic handgrip dynamometer at 53 and 60-64 years (two times and three times per hand respectively) and a Jamar electronic dynamometer at 69 years (two times per hand) were used[2]. A randomised repeated measurements cross-over trial found no statistically significant differences in values when comparing these two

devices[3]. For comparability with the other years, the first two values per hand at 60-64 years were used. In TwinsUK a Jamar hydraulic hand dynamometer was used (three times on the dominant hand)[4]. The maximum grip strength (kg) achieved in each study was used in analyses.

Chair rise speed: In NSHD, chair rise speed was measured at 53 and 60-64 years as the time taken for study participants to rise from a sitting to a standing position and sit down again ten times as fast as possible[5]. The same protocol was used at 69 years; however if study members were unable or unwilling to do ten chair rises, they were asked to do five instead. In TwinsUK, five chair rise speed tests were conducted. Chair rise speed was calculated as number of stands per minute and used as the outcome in analyses.

FEV_1: In NSHD, Forced Expiratory Volume (ml) in one second (FEV_1) was measured at 53 and 60-64 years using the Micromedical turbine electronic spirometer over two trials based on an NSHD protocol developed prior to the publication of the American Thoracic Society/European Respiratory Society guidelines[6,7]. The quality of spirometry was assessed by a nurse. At 69 years, FEV_1 was measured using the NDD Easy-On PC spirometer until three readings of similar values were achieved out of a maximum of five trials. In TwinsUK, FEV_1, was assessed according to the current American Thoracic Society/European Respiratory Society guidelines[6]. Three blows were performed and the maximum obtained value was retained. In NCDS, FEV_1 was assessed five times using a Vitalograph Micro hand-held spirometer[8]. The highest technically satisfactory value of up to three (two for NSHD) blows were used in analyses.

Cognitive performance

Cognitive performance was assessed by trained nurses at 53, 60-64 and 69 years in NSHD and at 50 years in NCDS using similar protocols.

Short term episodic memory: In NSHD, participants were shown 15 words and were asked to write down as many as possible from memory, in any order. This test was conducted three times, and the total number of words correctly recalled was summed (maximum 45)[9]. In NCDS, the number of words correctly recalled from 10 items read to the participants by a computer was recorded[10]. For comparability between the cohorts, a standardised measure (mean=0, SD=1) was used in analyses.

Mental speed: Mental speed was assessed using a letter cancellation test[9,10]. Briefly, participants were asked to cross out as many target letters (P and W) embedded among non-target letters as quickly and accurately as possible in one minute. The position reached at the end of the minute was used as an outcome in analyses.

Covariates

In each cohort, weight and height were measured using standard protocols and BMI was calculated as weight(kg)/(height(m))^2. Current smoking status was self-reported and categorised as current, ex-smoker or never smoker. Occupational social class was collected in NSHD and NCDS based on the Registrar General Classification. Income was assessed in TwinsUK with eight categories ranging from ≤£10,000 to ≥£100,000.

Supplementary table 1. Association between AgeAccelHannum and age-related performance

		Combined sex					Male only*				Female only			
	**Outcome**	**N**	**Coefficient (95% CI)**	***p*, I^2, $p_{heterogeneity}$**	**P non-linear**	p_{sex}	**N**	**Coefficient (95% CI)**	***p*, I^2, $p_{heterogeneity}$**	**P non-linear**	**N**	**Coefficient (95% CI)**	***p*, I^2, $p_{heterogeneity}$**	**P non-linear**
Overall (NSHD at 53y)	Grip strength (kg)	1,442	-0.10 (-0.22, 0.02)	0.11 0.0%, 0.50	0.34	-	-	-	-	-	805	-0.05 (-0.18, 0.09)	0.51, 0.0%, 0.82	0.77
	Chair rise speed (stands/m)	1,388	-0.123 (-0.248, 0.002)	0.05, 0.0%, 0.75	0.37	-	-	-	-	-	772	-0.05 (-0.22, 0.12)	0.57 0.0%, 0.55	0.99
	FEV_1(ml)	1,685	-0.005 (-0.20, 0.011)	0.54, 62.4%, 0.07	0.25	0.08	743	-0.003 (-0.014, 0.007)	0.54, 0.0%, 0.94	0.35	942	-0.01 (-0.03, 0.01)	0.44, 73.7%, 0.02	0.33
	Standardised number of words recalled	1,588	-0.007 (-0.019, 0.005)	0.23, 0.0%, 0.43	0.97	0.53	752	-0.004 (-0.021, 0.013)	0.63, 0.0%, 0.38	0.27	836	-0.01 (-0.03, 0.01)	0.21, 0.0%, 0.65	0.85
	Total letters scanned in one minute	1,602	-0.43 (-1.37, 0.51)	0.37, 0.0%, 0.94	0.15	0.86	761	-0.53 (-1.83, 0.77)	0.42, 0.0%, 0.43	0.78	841	-0.37 (-1.73, 0.98)	0.59, 0.0%, 0.65	0.08
Overall (NSHD at 60-64y)	Grip strength (kg)	738	-0.08 (-0.23, 0.07)	0.29, 0.0%, 0.57	0.72	-	-	-	-	-	424	-0.02 (-0.19, 0.16)	0.86, 0.0%, 0.96	0.25
	Chair rise speed (stands/m)	715	0.03 (-0.10, 0.16)	0.62, 0.0%, 0.38	0.36	-	-	-	-	-	406	-0.06 (-0.26, 0.13)	0.53 0.0%, 0.99	0.67
	FEV_1(ml)	1,009	-0.003 (-0.018, 0.013)	0.74, 54.6%, 0.11	0.79	0.37	443	-0.002 (-0.017, 0.013)	0.77, 0.0%, 0.89	0.77	586	-0.01 (-0.03, 0.01)	0.59, 64.9%, 0.06	0.90
	Standardised number of words recalled	880	-0.01 (-0.02, 0.01)	0.39 0.0%, 0.43	0.94	0.30	440	<0.001 (-0.022, 0.021)	0.97, 0.0%, 0.43	0.73	447	-0.02 (-0.04, 0.01)	0.21, 0.0%, 0.49	0.89
	Total letters scanned in one minute	894	0.27 (-0.97, 1.50)	0.67, 0.0%, 0.66	0.04	0.83	443	0.21 (-3.06, 3.48)	0.90, 33.4%, 0.22	0.38	451	-0.50 (-2.38, 1.34)	0.61, 0.0%, 0.59	0.54
NSHD 53y	Grip strength (kg)	1,323	-0.12 (-0.26, 0.02)	0.09	0.12	0.37	637	-0.18 (-0.41, 0.05)	0.12	0.23	686	-0.05 (-0.21, 0.10)	0.50	0.72
	Chair rise speed (stands/m)	1,287	-0.12 (-0.25, 0.01)	0.07	0.13	0.20	616	-0.20 (-0.39, -0.02)	0.03	0.07	671	-0.03 (-0.21, 0.14)	0.71	0.93
	FEV_1 (ml)	1,326	-0.009 (-0.016, -0.002)	0.01	0.03	0.11	632	-0.003 (-0.014, 0.008)	0.57	0.56	694	-0.014 (-0.023, -0.006)	<0.001	<0.001

	Standardised number of words recalled	1,348	-0.010 (-0.022, 0.004)	0.16	0.37	0.63	640	-0.006 (-0.025, 0.011)	0.48	0.42	708	-0.013 (-0.031, 0.006)	0.18	0.13
	Total letters scanned in one minute	1,364	-0.42 (-1.39, 0.55)	0.39	0.21	0.97	650	-0.42 (-1.75, 0.91)	0.53	0.65	714	-0.47 (-1.88, 0.95)	0.52	0.18
NSHD 60-64y	Grip strength (kg)	619	-0.10 (-0.29, 0.08)	0.28	0.54	0.49	314	-0.16 (-0.44, 0.12)	0.28	0.53	305	-0.01 (-0.24, 0.22)	0.84	0.29
	Chair rise speed (stands/m)	614	0.05 (-0.09, 0.18)	0.50	0.52	0.28	309	0.11 (-0.07, 0.28)	0.23	0.12	305	-0.04 (-0.25, 0.17)	0.69	0.84
	FEV_1(ml)	650	-0.004 (-0.015, 0.006)	0.41	0.49	0.56	332	-0.002 (-0.018, 0.014)	0.82	0.93	318	-0.008 (-0.021, 0.004)	0.20	0.18
	Standardised number of words recalled	648	-0.01 (-0.03, 0.01)	0.27	0.34	0.38	329	-0.004 (-0.027, 0.019)	0.76	0.90	319	-0.02 (-0.05, 0.01)	0.16	0.17
	Total letters scanned in one minute	656	0.36 (-0.95, 1.67)	0.59	0.11	0.19	332	1.08 (-0.63, 2.78)	0.22	0.17	324	-0.72 (-2.77, 1.33)	0.49	0.61
NCDS	FEV_1(ml)	239	-0.02 (-0.05, 0.01)	0.13	0.18	0.37	111	-0.01 (-0.05, 0.04)	0.81	0.95	128	-0.029 (-0.062, 0.003)	0.08	0.12
	Standardised number of words recalled	240	0.01 (-0.03, 0.04)	0.73	0.59	0.56	112	0.02 (-0.04, 0.08)	0.47	0.77	128	-0.001 (-0.048, 0.045)	0.96	0.45
	Total letters scanned in one minute	238	-0.55 (-4.34, 3.24)	0.78	0.81	0.38	111	-3.00 (-9.29, 3.29)	0.35	0.34	127	0.68 (-4.04, 5.39)	0.78	0.94
TwinsUK	Grip strength (kg)	119	-0.02 (-0.28, 0.24)	0.88	0.85	-	-	-	-	-	119	-0.02 (-0.28, 0.24)	0.88	0.85
	Chair rise speed (stands/m)	101	-0.21 (-0.78, 0.35)	0.46	0.93	-	-	-	-	-	101	-0.21 (-0.78, 0.35)	0.46	0.93
	FEV_1(ml)	120	0.01 (-0.01, 0.03)	0.19	0.46	-	-	-	-	-	120	0.01 (-0.01, 0.03)	0.19	0.46

All models adjusted for sex and age. Random effects accounting for twin pair was used in TwinsUK. Coefficient is the **c**hange in mean outcome per 1 year increase in AgeAccelHannum *Sex-stratified analyses for males is missing since TwinsUK only had female participants

Supplementary table 2. Association between AgeAccelHorvath and age-related performance

		Combined sex					Male only*				Female only			
	**Outcome**	**N**	**Coefficient (95% CI)**	***p*, I^2, p$_{heterogeneity}$**	**P non-linear**	***p*$_{sex}$**	**N**	**Coefficient (95% CI)**	***p*, I^2, p$_{heterogeneity}$**	**P non-linear**	**N**	**Coefficient (95% CI)**	***p*, I^2, p$_{heterogeneity}$**	**P non-linear**
Overall (NSHD at 53y)	Grip strength (kg)	1,442	-0.02 (-0.25, 0.21)	0.85 62.3%, 0.10	0.45	-	-	-	-	-	805	0.001 (-0.176, 0.178)	0.99, 34.2%, 0.22	0.60
	Chair rise speed (stands/m)	1,388	-0.08 (-0.20, 0.05)	0.25 0.0%, 0.61	0.75	-	-	-	-	-	772	-0.12 (-0.29, 0.06)	0.19, 0.0%, 0.50	0.80
	FEV$_1$(ml)	1,685	0.001 (-0.012, 0.014)	0.99, 53.6%, 0.12	0.04	0.01	743	0.007 (-0.004, 0.017)	0.22, 0.0%, 0.72	0.20	942	-0.004 (-0.024, 0.016)	0.69, 73.2%, 0.02	0.26
	Standardised number of words recalled	1,588	-0.001 (-0.013, 0.011)	0.89, 0.0%, 0.53	0.25	0.94	752	-0.001 (-0.018, 0.016)	0.90, 0.0%, 0.42	0.81	836	-0.001 (-0.018, 0.016)	0.91, 0.0%, 0.86	0.07
	Total letters scanned in one minute	1,602	-0.63 (-1.58, 0.33)	0.20 0.0%, 0.79	0.46	0.58	761	-0.18 (-2.64, 2.27)	0.88 24.0%, 0.25	0.40	841	-0.77 (-2.14, 0.60)	0.27, 0.0%, 0.50	0.82
Overall (NSHD at 60-64y)	Grip strength (kg)	738	0.01 (-0.14, 0.16)	0.86, 5.7%, 0.30	0.19	-	-	-	-	-	425	0.02 (-0.16, 0.19)	0.84, 11.9%, 0.29	0.03
	Chair rise speed (stands/m)	715	0.05 (-0.08, 0.17)	0.46, 0.0%, 0.97	0.69	-	-	-	-	-	403	0.02 (-0.16, 0.20)	0.86, 0.0%, 0.87	0.14
	FEV$_1$(ml)	1,009	<0.001 (-0.014, 0.015)	0.46, 55.4%, 0.11	0.24	0.89	443	-0.003 (-0.018, 0.011)	0.65, 0.0%, 0.83	0.17	566	<-0.000 (-0.017, 0.018)	0.98, 60.6%, 0.08	0.49
	Standardised number of words recalled	888	0.012 (-0.003, 0.027)	0.12, 0.0%, 0.86	0.52	0.49	441	0.017 (-0.004, 0.038)	0.10, 0.0%, 0.88	0.66	447	0.005 (-0.017, 0.028)	0.64, 0.0%, 0.88	0.73
	Total letters scanned in one minute	894	0.71 (-0.46, 1.87)	0.24, 0.0%, 0.60	0.40	0.05	443	1.76 (0.20, 3.33)	0.03, 0.0%, 0.74	0.40	451	-0.70 (-2.44, 1.03)	0.43, 0.0%, 0.46	0.95
NSHD 53y	Grip strength (kg)	1,323	-0.12 (-0.26, 0.02)	0.10	0.26	0.49	637	-0.17 (-0.40, 0.07)	0.16	0.35	686	-0.07 (-0.23, 0.09)	0.41	0.58
	Chair rise speed (stands/m)	1,287	-0.08 (-0.21, 0.05)	0.21	0.21	0.45	616	-0.03 (-0.22, 0.16)	0.72	0.86	671	-0.14 (-0.32, 0.05)	0.14	0.09
	FEV$_1$ (ml)	1,326	-0.001 (-0.008, 0.006)	0.71	0.09	0.02	632	0.007 (-0.004, 0.019)	0.20	0.18	694	-0.010 (-0.019, -0.002)	0.02	0.03

	Standardised number of words recalled	1,348	-0.002 (-0.015, 0.011)	0.72	0.70	0.92	640	-0.003 (-0.021 (-0.015)	0.72	0.88	708	-0.002 (-0.021, 0.017)	0.86	0.31
	Total letters scanned in one minute	1,364	-0.67 (-1.66, 0.33)	0.19	0.37	0.91	650	-0.73 (-2.09, 0.63)	0.29	0.56	714	-0.61 (-2.05, 0.84)	0.41	0.61
NSHD 60-64y	Grip strength (kg)	619	-0.04 (-0.22, 0.14)	0.65	0.30	0.87	314	-0.03 (-0.30, 0.24)	0.83	0.52	305	-0.06 (-0.28, 0.16)	0.59	0.36
	Chair rise speed (stands/m)	614	0.05 (-0.08, 0.17)	0.47	0.69	0.63	309	0.07 (-0.09, 0.24)	0.39	0.48	305	0.01 (-0.18, 0.20)	0.92	0.96
	FEV_1(ml)	650	-0.003 (-0.013, 0.007)	0.57	0.42	0.80	332	-0.004 (-0.020, 0.012)	0.62	0.33	318	-0.001 (-0.013, 0.011)	0.83	0.66
	Standardised number of words recalled	648	0.01 (-0.01, 0.03)	0.14	0.31	0.60	329	0.02 (-0.01, 0.04)	0.15	0.33	319	0.01 (-0.02, 0.03)	0.63	0.86
	Total letters scanned in one minute	656	0.82 (-0.42, 2.06)	0.20	0.43	0.10	332	1.68 (0.03, 3.31)	0.04	0.11	324	-0.41 (-2.32, 1.49)	0.67	0.83
NCDS	FEV_1(ml)	239	-0.01 (-0.04, 0.01)	0.26	0.53	0.35	111	0.0004 (-0.0365, 0.0373)	0.98	0.99	128	-0.02 (-0.05, 0.01)	0.14	0.34
	Standardised number of words recalled	240	0.01 (-0.02, 0.04)	0.59	0.38	0.62	112	0.02 (-0.04, 0.08)	0.47	0.60	128	0.003 (-0.040, 0.045)	0.91	0.58
	Total letters scanned in one minute	238	-0.17 (-3.61, 3.27)	0.92	0.06	0.17	111	2.67 (-2.99, 8.33)	0.36	0.02	127	-2.17 (-6.43, 2.08)	0.32	0.43
TwinsUK	Grip strength (kg)	119	0.12 (-0.13, 0.37)	0.35	0.06	-	-	-	-	-	119	0.12 (-0.13, 0.37)	0.35	0.06
	Chair rise speed (stands/m)	101	0.06 (-0.47, 0.59)	0.83	0.39	-	-	-	-	-	101	0.06 (-0.47, 0.59)	0.83	0.39
	FEV_1(ml)	120	0.016 (-0.002, 0.035)	0.09	0.96	-	-	-	-	-	120	0.016 (-0.002, 0.035)	0.09	0.96

All models adjusted for sex and age. Random effects accounting for twin pair was used in TwinsUK. Coefficient is the change in mean outcome per 1 year increase in AgeAccelHorvath *Sex-stratified analyses for males is missing since TwinsUK only had female participants

Supplementary table 3. Association between AgeAccelPheno and age-related performance

		Combined sex					Male only*				Female only			
	**Outcome**	**N**	**Coefficient (95% CI)**	***p*, I^2, $p_{heterogeneity}$**	**$P_{non-linear}$**	**p_{sex}**	**N**	**Coefficient (95% CI)**	***p*, I^2, $p_{heterogeneity}$**	**$P_{non-linear}$**	**N**	**Coefficient (95% CI)**	***p*, I^2, $p_{heterogeneity}$**	**$P_{non-linear}$**
Overall (NSHD at 53y)	Grip strength (kg)	1,442	-0.12 (-0.22, -0.02)	0.02 12.2%, 0.29	0.03	-	-	-	-	-	805	-0.092 (-0.185, <0.000)	0.05, 0.0%, 0.50	0.25
	Chair rise speed (stands/m)	1,388	-0.20 (-0.41, 0.01)	0.07 45.9%, 0.17	0.98	-	-	-	-	-	772	-0.20 (-0.41, <0.000)	0.05, 38.9%, 0.20	0.99
	FEV_1(ml)	1,685	-0.01 (-0.02, -0.01)	<0.001 0.0%, 0.52	0.19	0.93	743	-0.014 (-0.030, 0.002)	0.10, 53.2%, 0.14	0.53	942	-0.01 (-0.02, -0.01)	<0.001 0.0%, 0.88	0.29
	Standardised number of words recalled	1,588	-0.01 (-0.03, 0.01)	0.38, 58.9%, 0.12	0.42	0.81	752	-0.01 (-0.03, 0.01)	0.40 46.7%, 0.17	0.46	836	-0.012 (-0.024, <-0.000)	0.05, 0.0%, 0.43	0.68
	Total letters scanned in one minute	1,602	-0.97 (-1.65, -0.29)	0.005, 0.0%, 0.89	0.09	0.80	761	-1.05 (-2.02, -0.08)	0.03 0.0%, 0.48	0.65	841	-0.88 (-1.83, 0.08)	0.07, 0.0%, 0.52	0.06
Overall (NSHD at 60-64y)	Grip strength (kg)	738	-0.02 (-0.13, 0.08)	0.69, 0.0%, 0.77	0.43	-	-	-	-	-	425	0.02 (-0.11, 0.13)	0.79, 0.0%, 0.38	0.88
	Chair rise speed (stands/m)	715	-0.17 (-0.48, 0.14)	0.29 70.7%, 0.07	0.92	-	-	-	-	-	406	-0.17 (-0.10, 0.13)	0.31, 70.8%, 0.06	0.83
	FEV_1(ml)	1,009	-0.01 (-0.02, 0.01)	<0.001, 0.0%, 0.40	0.36	0.18	443	-0.016 (-0.028, -0.004)	0.01, 11.3%, 0.29	0.78	566	-0.008 (-0.015, -0.001)	0.03, 10.9%, 0.53	0.64
	Standardised number of words recalled	888	-0.01 (-0.02, 0.01)	0.31, 10.3%, 0.29	0.60	0.79	441	-0.01 (-0.03, 0.01)	0.39, 15.1%, 0.29	0.64	447	-0.01 (-0.02, 0.01)	0.52, 0.0%, 0.71	0.56
	Total letters scanned in one minute	894	-0.28 (-1.14, 0.59)	0.53, 0.0%, 0.46	0.65	0.27	443	-0.73 (-1.96, 0.48)	0.24, 0.0%, 0.36	0.86	451	0.24 (-0.99, 1.47)	0.70, 0.0%, 0.99	0.92
NSHD 53y	Grip strength (kg)	1,323	-0.15 (-0.25, -0.05)	<0.001	<0.001	0.45	637	-0.19 (-0.37, -0.02)	0.03	0.01	686	-0.113 (-0.223, -0.003)	0.04	0.08
	Chair rise speed (stands/m)	1,287	-0.13 (-0.23, -0.04)	0.01	0.01	0.87	616	-0.12 (-0.26, 0.02)	0.09	0.13	671	-0.14 (-0.27, -0.01)	0.03	0.04
	FEV_1 (ml)	1,326	-0.01 (-0.02, -0.01)	<0.001	<0.001	0.44	632	-0.008 (-0.016, 0.0002)	0.06	0.12	694	-0.01 (-0.02, -0.01)	<0.001	<0.001

	Standardised number of words recalled	1,348	-0.02 (-0.03, -0.01)	<0.001	<0.001	0.71	640	-0.018 (-0.031, -0.005)	0.01	0.03	708	-0.014 (-0.027, -0.001)	0.03	0.08
	Total letters scanned in one minute	1,364	-0.95 (-1.66, -0.25)	0.01	0.01	0.98	650	-0.95 (-1.96, 0.06)	0.07	0.15	714	-0.97 (-1.96, 0.02)	0.06	0.04
NSHD 60-64y	Grip strength (kg)	619	-0.01 (-0.14, 0.12)	0.89	0.85	0.34	314	-0.07 (-0.29, 0.14)	0.49	0.67	304	0.06 (-0.09, 0.21)	0.43	0.55
	Chair rise speed (stands/m)	614	-0.05 (-0.14, 0.05)	0.34	0.44	0.81	309	-0.05 (-0.1, 0.08)	0.42	0.53	305	-0.03 (-0.17, 0.10)	0.62	0.83
	FEV_1(ml)	650	-0.008 (-0.016, -0.001)	0.03	0.09	0.29	332	-0.012 (-0.024, 0.001)	0.06	0.15	318	-0.004 (-0.012, 0.004)	0.34	0.48
	Standardised number of words recalled	648	-0.010 (-0.023, 0.003)	0.12	0.22	0.64	329	-0.013 (-0.031, 0.004)	0.14	0.30	319	-0.007 (-0.025, 0.011)	0.46	0.70
	Total letters scanned in one minute	656	-0.15 (-1.08, 0.77)	0.74	0.82	0.42	332	-0.52 (-1.82, 0.78)	0.43	0.70	324	0.24 (-1.07, 1.56)	0.72	0.17
NCDS	FEV_1(ml)	239	-0.019 (-0.035, -0.002)	0.03	0.08	0.35	111	-0.026 (-0.048, -0.004)	0.02	0.08	128	-0.010 (-0.034, 0.014)	0.40	0.56
	Standardised number of words recalled	240	0.005 (-0.019, 0.029)	0.70	0.73	0.75	112	0.008 (-0.027, 0.043)	0.65	0.34	128	0.001 (-0.033, 0.034)	0.98	0.88
	Total letters scanned in one minute	238	-1.14 (-3.59, 1.31)	0.36	0.64	0.32	111	-2.26 (-5.76, 1.23)	0.39	0.21	127	0.21 (-3.23, 3.65)	0.90	0.45
TwinsUK	Grip strength (kg)	119	-0.04 (-0.21, 0.13)	0.63	0.56	-	-	-	-	-	119	-0.04 (-0.21, 0.13)	0.63	0.56
	Chair rise speed (stands/m)	101	-0.37 (-0.70, -0.04)	0.03	0.29	-	-	-	-	-	101	-0.37 (-0.70, -0.04)	0.03	0.29
	FEV_1(ml)	120	-0.015 (-0.028, -0.003)	0.02	0.17	-	-	-	-	-	120	-0.015 (-0.028, -0.003)	0.02	0.17

All models adjusted for sex and age. Random effects accounting for twin pair was used in TwinsUK. Coefficient is the change in mean outcome per 1 year increase in AgeAccelPheno *Sex-stratified analyses for males is missing since TwinsUK only had female participants

Supplementary table 4. Association between AgeAccelGrim and age-related performance

		Combined sex					Male only*				Female only			
	**Outcome**	**N**	**Coefficient (95% CI)**	***p*, I^2, $p_{heterogeneity}$**	**P non-linear**	p_{sex}	**N**	**Coefficient (95% CI)**	***p*, I^2, $p_{heterogeneity}$**	**P non-linear**	**N**	**Coefficient (95% CI)**	***p*, I^2, $p_{heterogeneity}$**	**P non-linear**
Overall (NSHD at 53y)	Grip strength (kg)	1,442	-0.03<br>(-0.14, 0.07)	0.54<br>0.0%,<br>0.67	0.98	-	-	-	-	-	805	-0.10<br>(-0.22, 0.02)	0.12<br>0.0%,<br>0.98	0.96
	Chair rise speed (stands/m)	1,388	-0.43<br>(-0.99, 0.14)	0.14<br>70.0%,<br>0.07	0.68	-	-	-	-	-	772	-0.45<br>(-0.94, 0.04)	0.07,<br>61.7%,<br>0.12	0.82
	FEV_1(ml)	1,685	-0.03<br>(-0.05, -0.01)	0.002<br>79.5%,<br>0.01	0.60	0.77	743	-0.02<br>(-0.03, -0.01)	<0.001<br>0.0%,<br>0.47	0.97	942	-0.03<br>(-0.06, -0.01)	0.002,<br>76.5%,<br>0.01	0.46
	Standardised number of words recalled	1,588	-0.03<br>(-0.05, -0.02)	<0.001,<br>30.4%,<br>0.23	0.10	0.47	752	-0.02<br>(-0.06, 0.02)	0.26<br>67.5%,<br>0.08	0.60	836	-0.04<br>(-0.05, -0.02)	<0.001<br>0.0%,<br>0.83	0.07
	Total letters scanned in one minute	1,602	-2.05<br>(-2.81, -1.29)	<0.001<br>0.0%,<br>0.99	0.83	0.33	761	-2.44<br>(-3.49, -1.40)	<0.001<br>0.0%,<br>0.59	0.81	841	-1.69<br>(-2.80, -0.58)	0.003<br>0.0%,<br>0.58	0.92
Overall (NSHD at 60-64y)	Grip strength (kg)	738	-0.22<br>(-0.37, -0.06)	0.01,<br>0.0%,<br>0.42	0.01	-	-	-	-	-	425	-0.11<br>(-0.29, 0.07)	0.24,<br>0.00%,<br>0.96	0.24
	Chair rise speed (stands/m)	715.96	-0.44<br>(-0.96, 0.08)	0.09<br>65.0%,<br>0.09	0.88	-	-	-	-	-	406	-0.44<br>(-0.98, -0.10)	0.11<br>66.0%,<br>0.08	0.79
	FEV_1(ml)	1,009	-0.04<br>(-0.05, -0.02)	<0.001,<br>76.4%,<br>0.01	0.44	0.76	443	<0.000<br>(-0.002, 0.003)	0.72,<br>0.0%,<br>0.52	0.72	566	-0.04<br>(-0.06, -0.02)	<0.001<br>68.2%,<br>0.04	0.10
	Standardised number of words recalled	888	-0.03<br>(-0.06, -0.01)	0.01,<br>61.0%,<br>0.01	0.77	0.66	441	0.002<br>(-0.003, 0.006)	0.51,<br>27.1%,<br>0.24	0.51	447	-0.03<br>(-0.05, -0.01)	0.002,<br>0.0%,<br>0.65	0.91
	Total letters scanned in one minute	894	-2.37<br>(-3.50, -1.25)	<0.001<br>0.0%,<br>0.81	0.87	0.45	443	-3.27<br>(-4.93, -1.61)	<0.001,<br>5.2%,<br>0.30	0.86	451	-1.27<br>(-2.94, 0.39)	0.13<br>0.0%,<br>0.41	0.30
NSHD 53y	Grip strength (kg)	1,323	-0.03<br>(-0.14, 0.09)	0.66	0.12	0.25	637	0.04<br>(-0.15, 0.23)	0.68	0.66	686	-0.10<br>(-0.22, 0.03)	0.15	0.06
	Chair rise speed (stands/m)	1,287	-0.21<br>(-0.32, -0.10)	<0.001	<0.001	0.29	616	-0.156<br>(-0.311, -0.001)	0.05	0.14	671	-0.27<br>(-0.42, -0.12)	<0.001	<0.001
	FEV_1 (ml)	1,326	-0.02<br>(-0.03, -0.02)	<0.001	<0.001	0.82	632	-0.02<br>(-0.03, -0.02)	<0.001	<0.001	694	-0.03<br>(-0.03, -0.02)	<0.001	<0.001

	Standardised number of words recalled	1,348	-0.04 (-0.05, -0.02)	<0.001	<0.001	0.92	640	-0.03 (-0.05, -0.02)	<0.001	<0.001	708	-0.04 (-0.05, -0.02)	<0.001	<0.001
	Total letters scanned in one minute	1,364	-2.05 (-2.84, -1.26)	<0.001	<0.001	0.25	650	-2.52 (-3.60, -1.44)	<0.001	<0.001	714	-1.60 (-2.76, -0.44)	0.01	0.03
NSHD 60-64y	Grip strength (kg)	619	-0.25 (-0.42, -0.07)	0.01	<0.001	0.17	314	-0.36 (-0.64, -0.08)	0.01	0.01	305	-0.11 (-0.32, 0.10)	0.31	0.47
	Chair rise speed (stands/m)	614	-0.25 (-0.38, -0.12)	<0.001	<0.001	0.26	309	-0.27 (-0.45, -0.09)	<0.001	0.01	205	-0.23 (-0.42, -0.04)	0.02	0.06
	FEV_1(ml)	650	-0.03 (-0.04, -0.02)	<0.001	<0.001	0.63	332	-0.04 (-0.05, -0.02)	<0.001	<0.001	318	-0.03 (-0.04, -0.02)	<0.001	<0.001
	Standardised number of words recalled	648	-0.04 (-0.06, -0.03)	<0.001	<0.001	0.15	329	-0.05 (-0.08, -0.03)	<0.001	<0.001	319	-0.03 (-0.05, -0.01)	0.02	0.07
	Total letters scanned in one minute	6756	-2.43 (-3.67, -1.19)	<0.001	<0.001	0.03	332	-3.65 (-5.32, -1.92)	<0.001	<0.001	324	-0.93 (-2.78, 0.92)	0.33	0.41
NCDS	FEV_1(ml)	239	-0.0016 (-0.034, 0.003)	0.10	0.19	0.82	111	-0.01 (-0.04, 0.01)	0.30	0.45	128	-0.02 (-0.04, 0.01)	0.18	0.39
	Standardised number of words recalled	240	-0.02 (-0.04, 0.01)	0.21	0.18	0.12	112	0.003 (-0.037, 0.043)	0.89	0.41	128	-0.040 (-0.078, -0.003)	0.04	0.11
	Total letters scanned in one minute	238	-2.07 (-4.82, 0.69)	0.14	0.34	0.62	111	-1.38 (-5.37, 2.62)	0.50	0.44	127	-2.73 (-6.55, 1.09)	0.16	0.33
TwinsUK	Grip strength (kg)	119	-0.10 (-0.42, 0.23)	0.55	0.28	-	-	-	-	-	119	-0.10 (-0.42, 0.23)	0.55	0.28
	Chair rise speed (stands/m)	101	-0.81 (-1.44, -0.18)	0.01	0.29	-	-	-	-	-	101	-0.81 (-1.44, -0.18)	0.01	0.29
	FEV_1(ml)	120	-0.06 (-0.08, -0.04)	<0.001	0.03	-	-	-	-	-	120	-0.06 (-0.08, -0.04)	<0.001	0.03

All models adjusted for sex and age. Random effects accounting for twin pair was used in TwinsUK. Coefficient is the change in mean outcome per 1 year increase in AgeAccelGrim *Sex-stratified analyses for males is missing since TwinsUK only had female participants

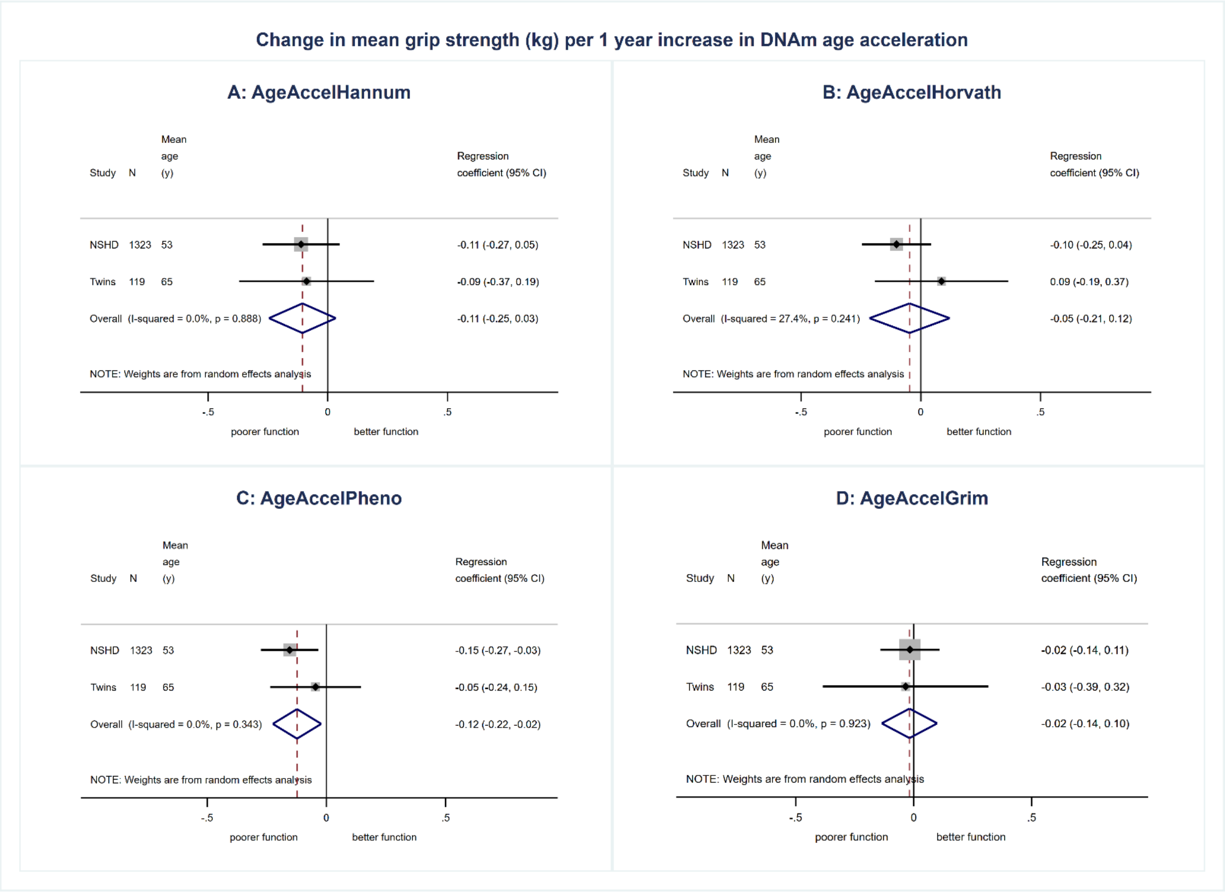

Supplementary figure 1. Association between (A) AgeAccelHannum (B) AgeAccelHorvath (C) AgeAccelPheno (D) AgeAccelGrim and grip strength adjusted for sex, age and cell composition

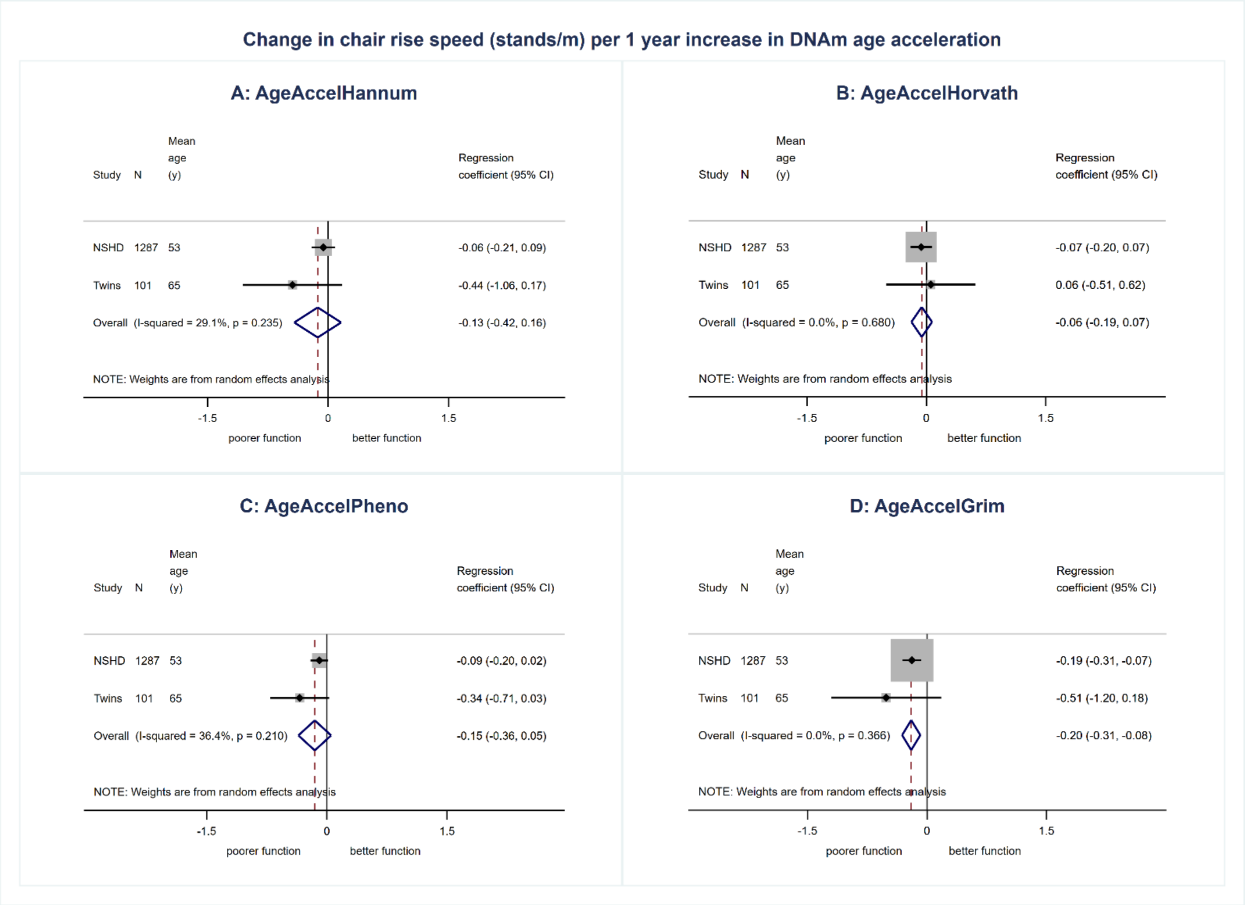

Supplementary figure 2. Association between (A) AgeAccelHannum (B) AgeAccelHorvath (C) AgeAccelPheno (D) AgeAccelGrim and chair rise speed adjusted for sex, age and cell composition

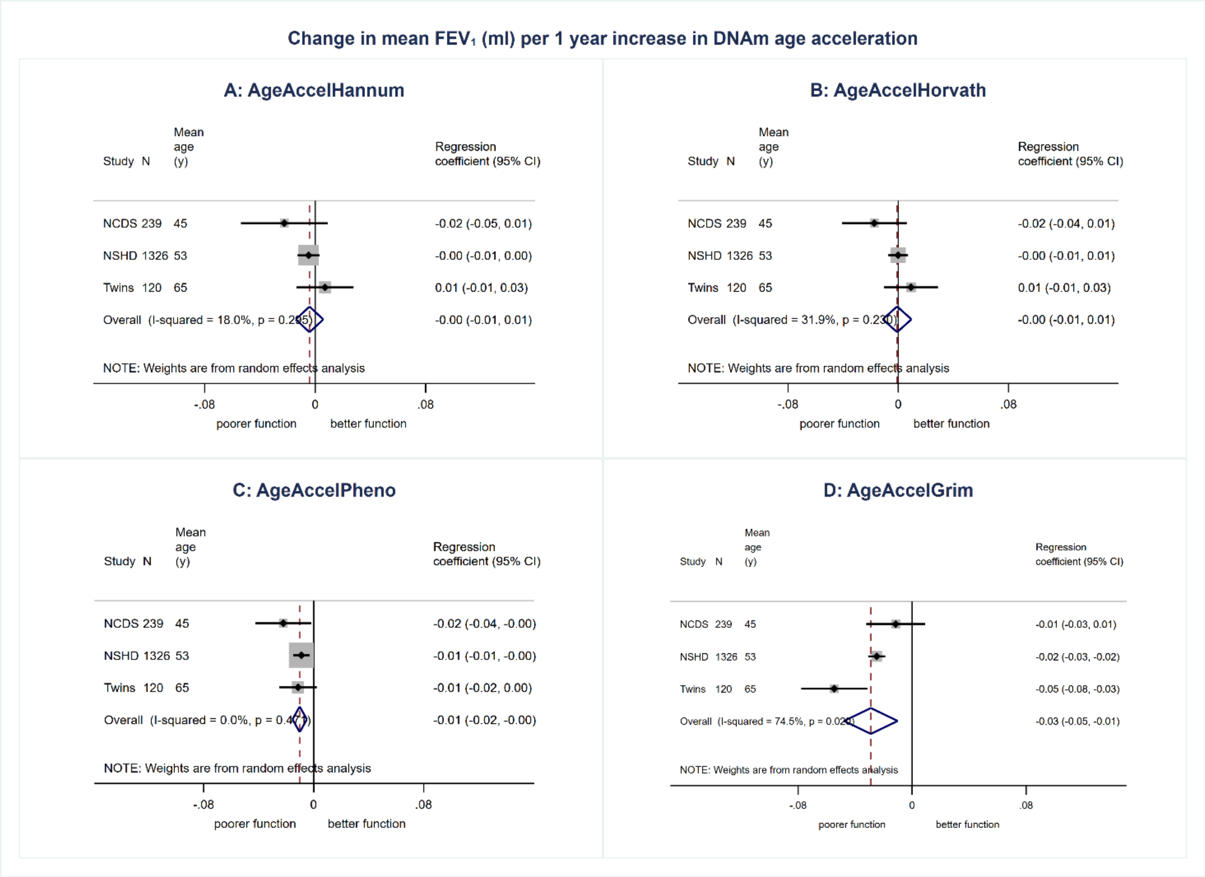

Supplementary figure 3. Association between (A) AgeAccelHannum (B) AgeAccelHorvath (C) AgeAccelPheno (D) AgeAccelGrim and FEV_1 adjusted for sex, age and cell composition

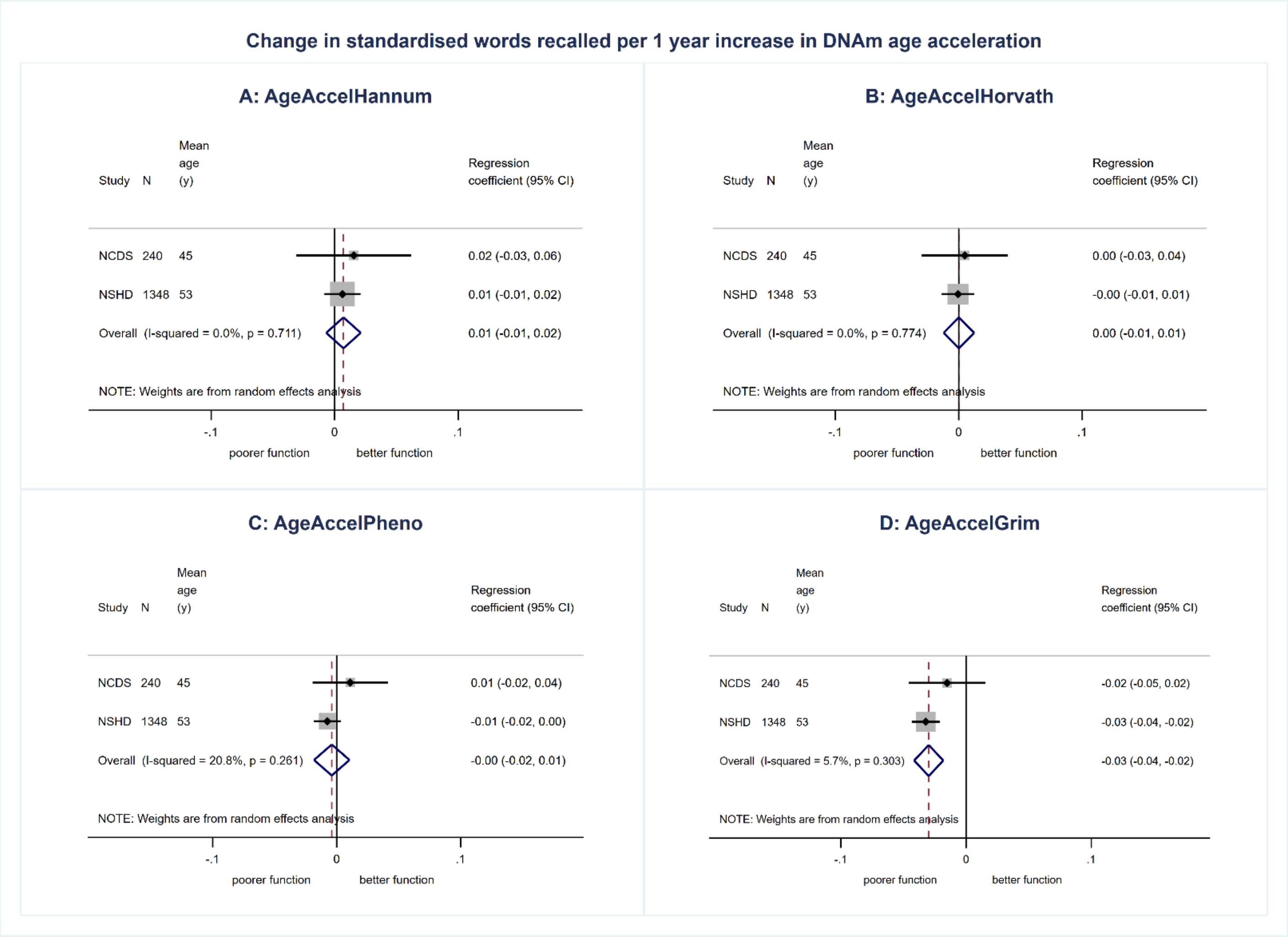

Supplementary figure 4. Association between (A) AgeAccelHannum (B) AgeAccelHorvath (C) AgeAccelPheno (D) AgeAccelGrim and standardised number of words recalled adjusted for sex, age and cell composition

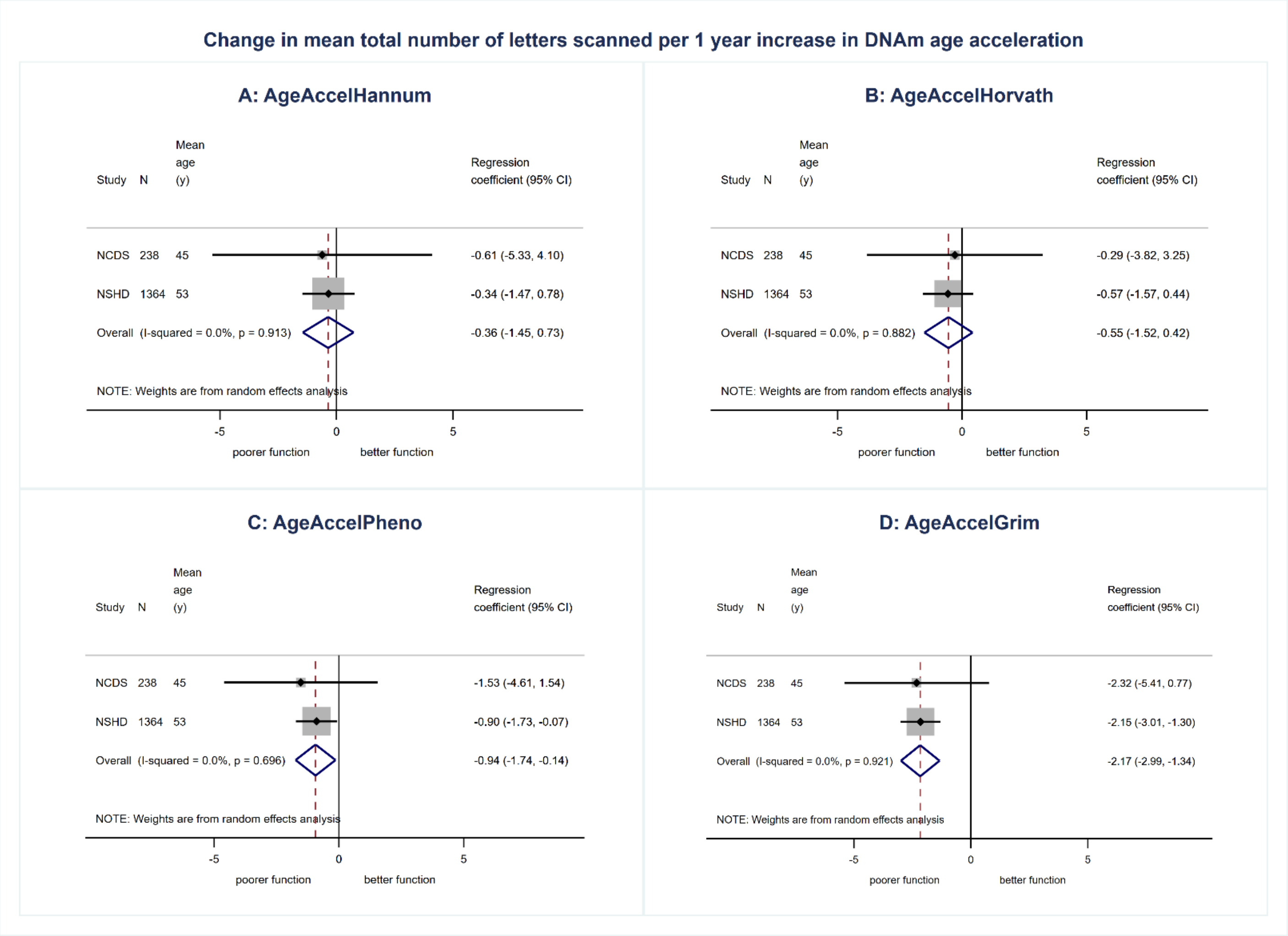

Supplementary figure 5. Association between (A) AgeAccelHannum (B) AgeAccelHorvath (C) AgeAccelPheno (D) AgeAccelGrim and total number of letters scanned adjusted for sex, age and cell composition

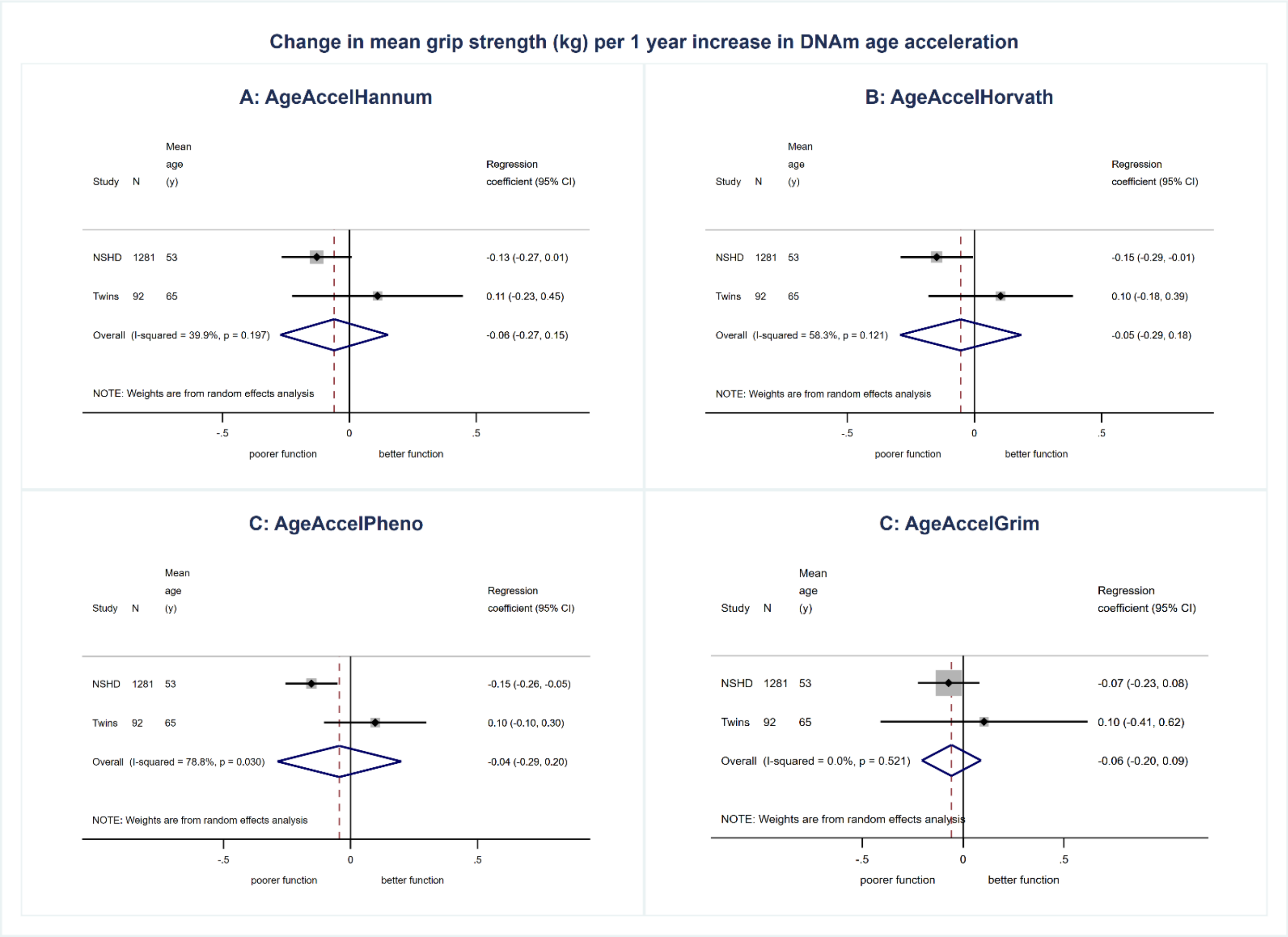

Supplementary figure 6. Association between (A) AgeAccelHannum (B) AgeAccelHorvath (C) AgeAccelPheno (D) AgeAccelGrim and grip strength adjusted for sex, age, BMI, height, smoking status, and social class (NSHD and NCDS) or income (TwinsUK)

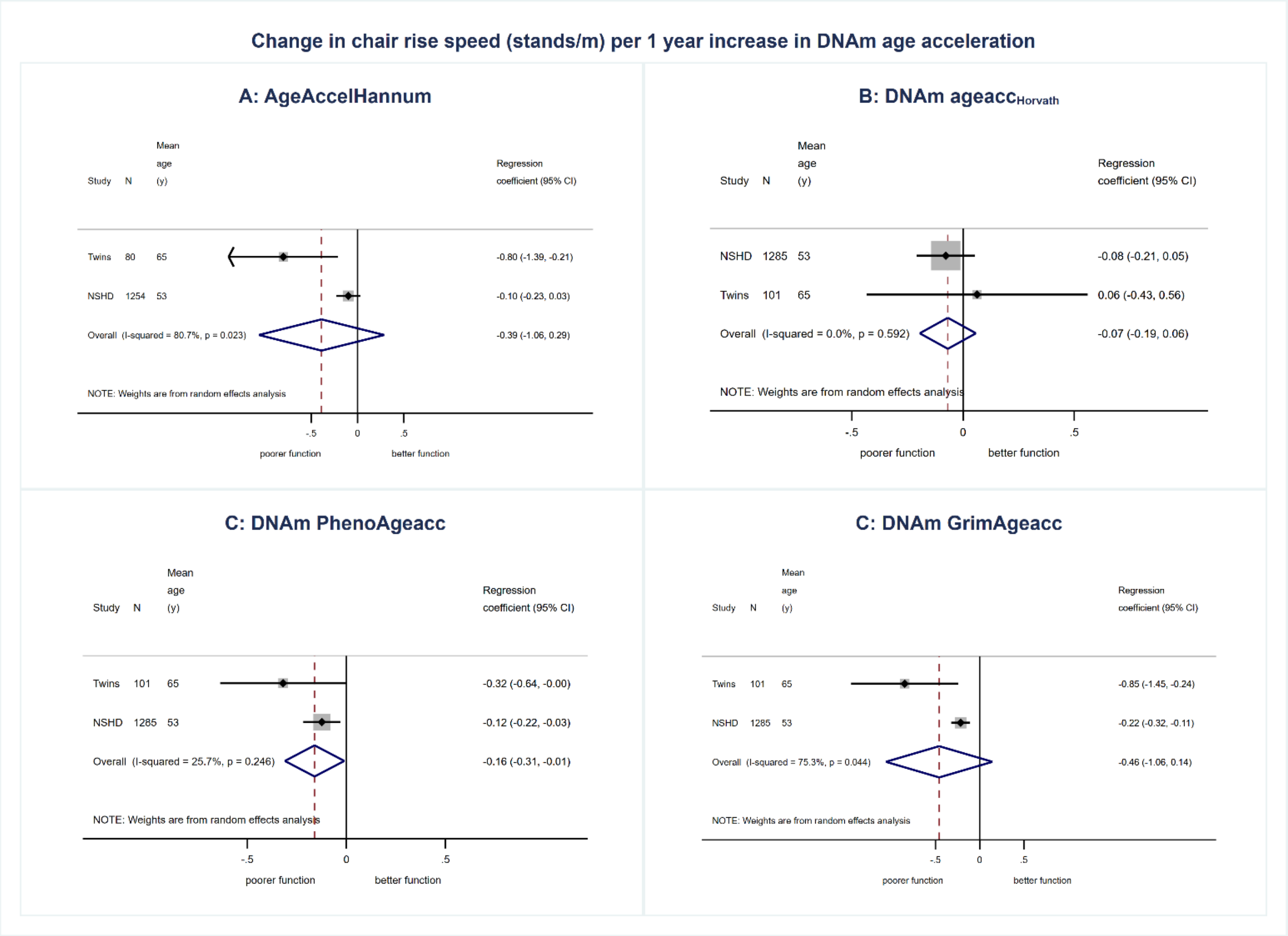

Supplementary figure 7. Association between (A) AgeAccelHannum (B) AgeAccelHorvath (C) AgeAccelPheno (D) AgeAccelGrim and chair rise speed adjusted for sex, age, BMI, height, smoking status and social class (NSHD and NCDS) or income (TwinsUK)

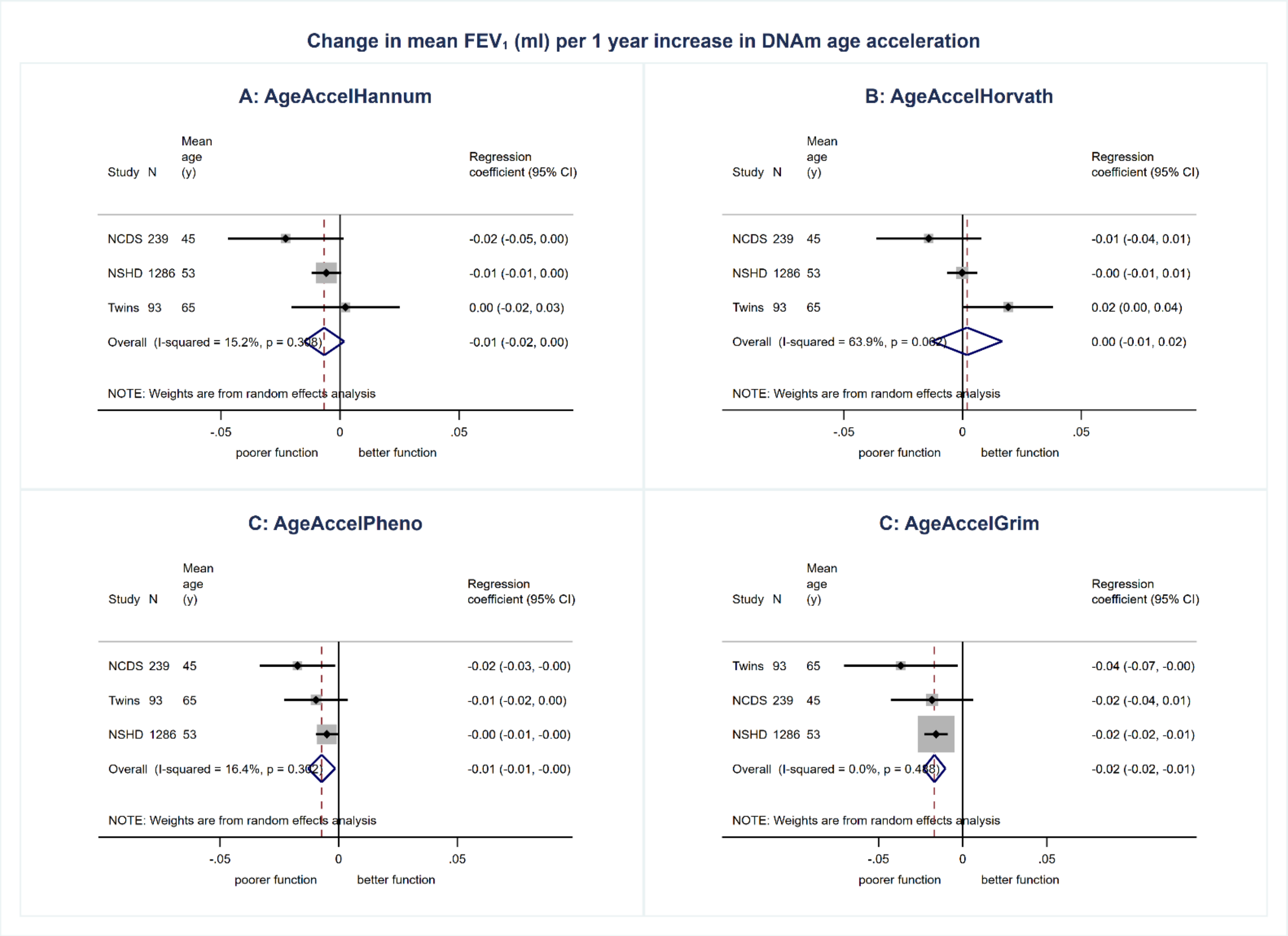

Supplementary figure 8. Association between (A) AgeAccelHannum (B) AgeAccelHorvath (C) AgeAccelPheno (D) AgeAccelGrim and FEV_1 adjusted for sex, age, BMI, height, smoking status and social class (NSHD and NCDS) or income (TwinsUK)

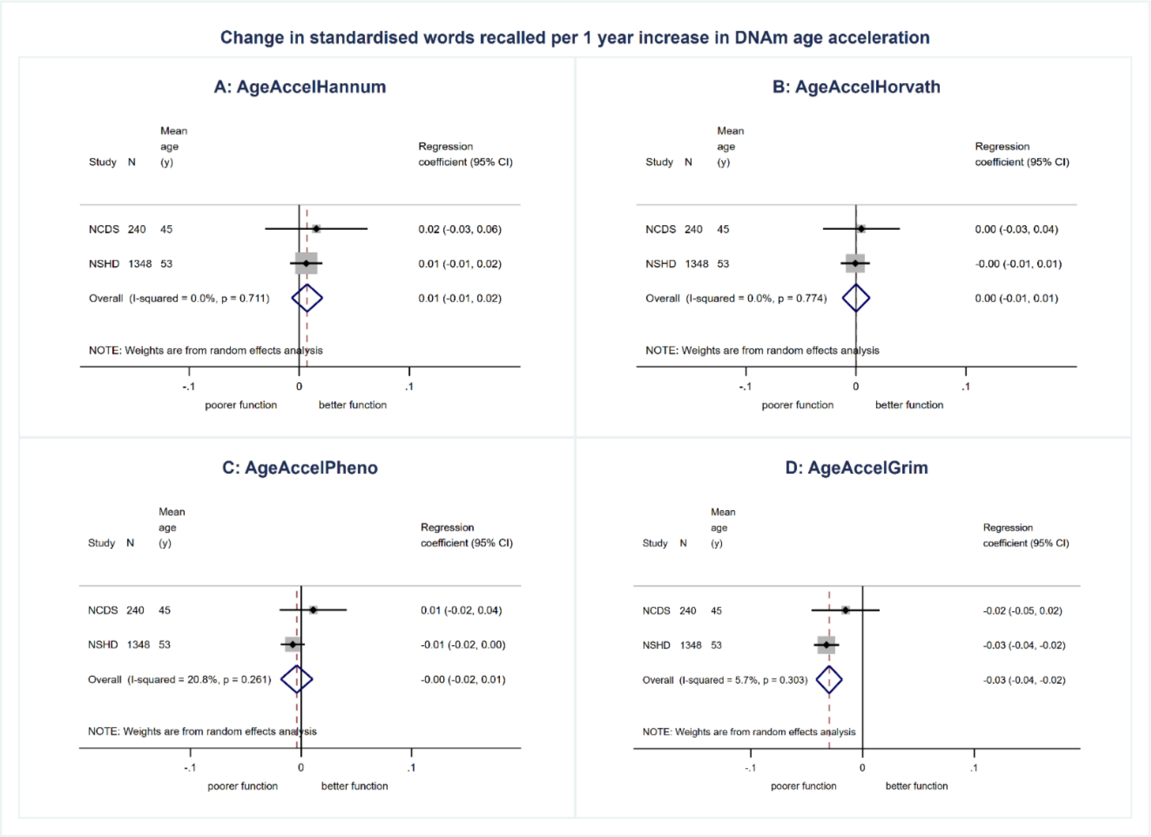

Supplementary figure 9. Association between (A) AgeAccelHannum (B) AgeAccelHorvath (C) AgeAccelPheno (D) AgeAccelGrim standardised total number of words recalled adjusted for sex, age, BMI, height, smoking status and social class (NSHD and NCDS) or income (TwinsUK)

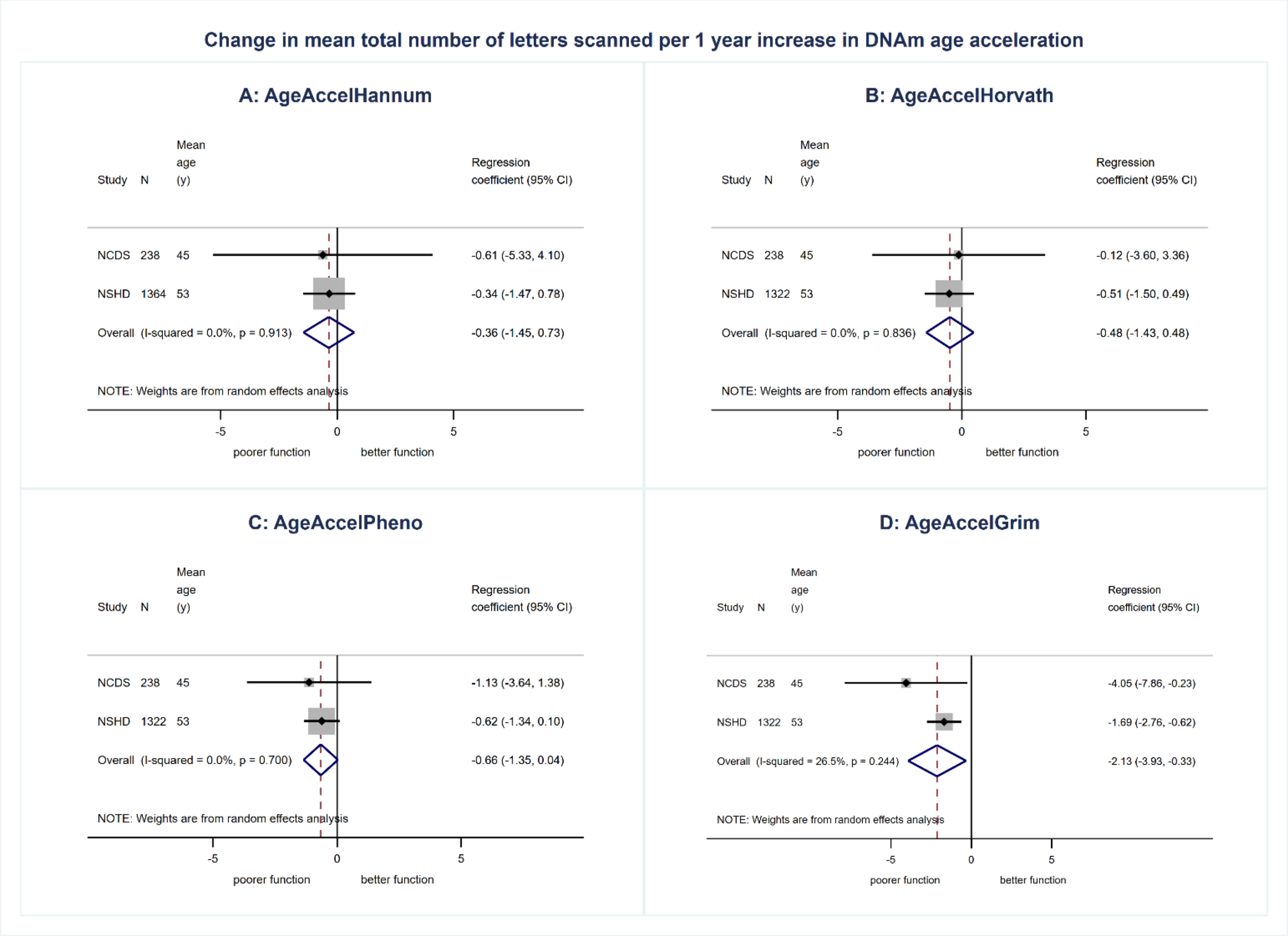

Supplementary figure 10. Association between (A) AgeAccelHannum (B) AgeAccelHorvath (C) AgeAccelPheno (D) AgeAccelGrim total number of letters scanned adjusted for sex, age, BMI, height, smoking status and social class (NSHD and NCDS) or income (TwinsUK)

Supplementary table 5. Summary of age-related performance in NSHD at 53, 60-64 and 69 years [mean(SD)]

		53y		60-64y		69y		Decline over time (per year)**
	**N***	**Male**	**Female**	**Male**	**Female**	**Male**	**Female**	**β(95%CI)**
Grip strength, kg	1080	48.7 (12.4)	27.9 (8.4)	45.3 (11.8)	25.9 (7.6)	40.2 (8.7)	24.0 (5.6)	-0.37 (-0.42, -0.33)
Chair rise speed, stands/min	1041	32.3 (9.9)	30.7 (9.4)	26.5 (7.2)	25.6 (8.1)	27.5 (8.4)	26.5 (8.5)	-0.32 (-0.37, -0.28)
FEV_1, ml	1060	3.32 (0.58)	2.34 (0.44)	3.06 (0.67)	2.12 (0.46)	2.97 (0.62)	2.06 (0.45)	-0.022 (-0.024, -0.021)
Number of words recalled	1087	23.83 (5.9)	25.0 (6.1)	23.4 (5.9)	25.0 (6.0)	21.4 (6.0)	23.0 (6.0)	-0.13 (-0.14, -0.10)
Total letters scanned	1109	276.9 (71.8)	294.6 (76.4)	261.0 (73.2)	276.2 (76.7)	256.3 (72.8)	269.5 (73.9)	-1.46 (-1.78, -1.19)

*N includes participants with performance measures from at least two time points and AgeAccel at 53 years;
**Estimates from linear mixed models adjusted for sex. All p-values <0.001

Supplementary table 6. Association between AgeAccel at 53 years and longitudinal change in performance (53-69 years) in NSHD adjusted for sex, age and cell composition at 53 years

	Grip strength, kg (n=1,362)		Chair rise speed, stands/min (n=1,334)		FEV_1, ml (n=1,359)		Number of words (n=1,358)		Total number of letters scanned (n=1,368)	
	Estimate (95% CI)	p-value	Estimate (95% CI)	p-value	Estimate (95% CI)	p-value	Estimate (95% CI)	p-value	Estimate (95% CI)	p-value
AgeAccelHannum	-0.09 (-0.21, 0.03)	0.15	-0.07 (-0.18, 0.04)	0.21	-0.004 (-0.012, 0.003)	0.26	0.004 (-0.079, 0.088)	0.92	-0.24 (-1.21, 0.73)	0.63
AgeAccelHorvath	-0.06 (-0.16, 0.05)	0.29	-0.08 (-0.18, 0.02)	0.12	0.002 (-0.005, 0.009)	0.63	-0.01 (-0.08, 0.07)	0.82	-0.21 (-1.09, 0.66)	0.63
AgeAccelPheno	-0.14 (-0.22, -0.05)	0.002	-0.09 (-0.17, -0.0003)	0.04	-0.009* (-0.014, -0.003)	<0.001	-0.064 (-0.126, -0.003)	0.04	-0.99 (-1.71,-0.27)	0.01
AgeAccelPhenoXtime					-0.0003** (-0.0006, <0.0000)	0.04				
AgeAccelGrim	0.004* (-0.115, 0.123)	0.94	-0.23 (-0.31, -0.14)	<0.001	-0.02* (-0.03,-0.02)	<0.001	-0.21 (-0.28,-0.15)	<0.001	-2.06 (-2.81,-1.32)	<0.001
AgeAccelGrimXtime	-0.02** (-0.02,-0.01)	<0.001			-0.001** (-0.001, -0.001)	<0.001				

Estimates represent average change in outcome over 16 years for a one year increase in AgeAccel at 53 years unless p-value from log-likelihood ratio test comparing models fit with an interaction term for time to models without the interaction term = ≤0.05 then:
*estimates represent the difference in outcome at 53 years for a one year increase at in AgeAccel at 53 years i.e. at the intercept
**estimates represent the difference in average change in the linear slope over time from 53 years.

Supplementary table 7. Association between AgeAccel at 53 years and longitudinal change in performance (53-69 years) in NSHD adjusted for sex, age, social class at 53 years and time-varying BMI, height and smoking status

	Grip strength, kg (*n*=1,317)		Chair rise speed, stands/min (*n*=1,294)		FEV_1, ml (*n*=1,318)		Number of words (*n*=1,317)		Total number of letters scanned (*n*=1,327)	
	Estimate (95% CI)	p-value	Estimate (95% CI)	p-value	Estimate (95% CI)	p-value	Estimate (95% CI)	p-value	Estimate (95% CI)	p-value
AgeAccelHannum	-0.102 (-0.203, -0.001)	0.05	-0.09 (-0.18, 0.01)	0.07	-0.006 (-0.012, <-0.001)	0.05	-0.05 (-0.12, 0.02)	0.14	-0.41 (-1.24, 0.43)	0.34
AgeAccelHorvath	-0.10 (-0.20, 0.01)	0.07	-0.07 (-0.17, 0.03)	0.18	0.002 (-0.004, 0.008)	0.62	0.01 (-0.06, 0.08)	0.85	-0.25 (-1.12, 0.61)	0.57
AgeAccelPheno	-0.13 (-0.21, -0.06)	<0.001	-0.06 (-0.13, 0.01)	0.09	-0.007 (-0.011, -0.002)	0.002	-0.06 (-0.11, -0.01)	0.02	-0.77 (-1.39, -0.14)	0.02
AgeAccelGrim	0.04* (-0.09, 0.17)	0.57	-0.19 (-0.29, -0.09)	<0.001	-0.02* (-0.02, -0.02)	<0.001	-0.16 (-0.22, -0.09)	<0.001	-1.70 (-2.55, -0.84)	<0.001
AgeAccelGrim Xtime	-0.017** (-0.025, -0.008)	<0.001			-0.0009** (-0.001, -0.0005)	<0.001				

Estimates represent average change in outcome over 16 years for a one year increase in AgeAccel at 53 years unless p-value from log-likelihood ratio test comparing models fit with an interaction term for time to models without the interaction term = ≤0.05 then:
*estimates represent the difference in outcome at 53 years for a one year increase at in AgeAccel at 53 years i.e. at the intercept
**estimates represent the difference in average change in the linear slope over time from 53 years.

Supplementary table 8. Association between change in AgeAccel and change in age-related performance between 53 years and 60-64y conditional on baseline performance at 53 years adjusted for sex, age and cell composition at 53 years

	ΔGrip strength, kg (*n*=435)		ΔChair rise, stands/min (*n*=418)		Δ FEV_1, ml (*n*=451)		ΔNumber of words recalled (*n*=456)		ΔTotal number of letters scanned (*n*=464)	
	Estimate (95% CI)	p-value	Estimate (95% CI)	p-value	Estimate (95% CI)	p-value	Estimate (95% CI)	p-value	Estimate (95% CI)	p-value
ΔAgeAccelHannum	0.13 (-0.14, 0.40)	0.34	0.03 (-0.16, 0.21)	0.77	0.004 (-0.009, 0.018)	0.54	-0.03 (-0.16, 0.10)	0.65	0.11 (-1.69, 1.92)	0.90
ΔAgeAccelHorvath	-0.04 (-0.27, 0.19)	0.73	-0.10 (-0.26, 0.06)	0.21	-0.003 (-0.014, 0.008)	0.58	0.06 (-0.05, 0.17)	0.31	1.07 (-0.46, 2.60)	0.17
ΔAgeAccelPheno	0.04 (-0.14, 0.22)	0.64	-0.11 (-0.24, 0.02)	0.09	0.004 (-0.005, 0.013)	0.43	0.03 (-0.06, 0.12)	0.49	0.42 (-0.81, 1.66)	0.50
ΔAgeAccelGrim	-0.20 (-0.36, 0.04)	0.02	-0.16 (-0.27, -0.04)	0.01	-0.01 (-0.02, 0.0001)	0.05	-0.04 (-0.12, 0.04)	0.34	-0.90 (-2.00, 0.21)	0.11

Supplementary table 9. Association between change in AgeAccel and change in age-related performance between 53 years and 60-64y conditional on baseline performance at 53 years adjusted for sex, age, social class, BMI, height and smoking status at 53 years

	ΔGrip strength, kg (*n*=428)		ΔChair rise, stands/min (*n*=414)		Δ FEV_1 , ml (*n*=443)		ΔNumber of words recalled (*n*=448)		ΔTotal number of letters scanned (*n*=459)	
	Estimate (95% CI)	p-value	Estimate (95% CI)	p-value	Estimate (95% CI)	p-value	Estimate (95% CI)	p-value	Estimate (95% CI)	p-value
ΔAgeAccelHannum	0.21 (-0.04, 0.47)	0.10	0.03 (-0.14, 0.20)	0.70	0.01 (-0.01, 0.02)	0.37	0.02 (-0.10, 0.14)	0.73	0.37 (-1.30, 2.05)	0.66
ΔAgeAccelHorvath	-0.10 (-0.33, 0.12)	0.38	-0.06 (-0.22, 0.09)	0.43	-0.01 (-0.02, 0.01)	0.32	0.08 (-0.02, 0.19)	0.13	0.97 (-0.54, 2.49)	0.21
ΔAgeAccelPheno	0.04 (-0.14, 0.21)	0.67	-0.12 (-0.24, 0.01)	0.06	0.004 (-0.005, 0.013)	0.37	0.06 (-0.02, 0.15)	0.14	0.24 (-0.96, 1.43)	0.69
ΔAgeAccelGrim	-0.10 (-0.26, 0.06)	0.21	-0.14 (-0.25, 0.03)	0.01	0.001 (-0.007, 0.010)	0.77	0.02 (-0.06, 0.10)	0.63	-0.73 (-1.83, 0.38)	0.20

References

1. Fortin J-P, Triche Jr TJ, Hansen KD. Preprocessing, normalization and integration of the Illumina HumanMethylationEPIC array with minfi. *Bioinformatics.* 2016;33(4):558-560.
2. Kuh D, Hardy R, Butterworth S, et al. Developmental origins of midlife grip strength: findings from a birth cohort study. *The Journals of Gerontology Series A: Biological Sciences and Medical Sciences.* 2006;61(7):702-706. doi:10.1093/gerona/61.7.702.
3. Kuh D, Hardy R, Blodgett J, Cooper R. Developmental factors associated with decline in grip strength from midlife to old age: a British birth cohort study. *bioRxiv.* 2018;72:327247. doi:10.1136/jech-2018-SSMabstracts.181.
4. Welch AA, Kelaiditi E, Jennings A, Steves CJ, Spector TD, MacGregor A. Dietary Magnesium Is Positively Associated With Skeletal Muscle Power and Indices of Muscle Mass and May Attenuate the Association Between Circulating C-Reactive Protein and Muscle Mass in Women. *Journal of Bone and Mineral Research.* 2016;31(2):317-325. doi:10.1002/jbmr.2692.
5. Kuh D, Bassey EJ, Butterworth S, Hardy R, Wadsworth ME. Grip strength, postural control, and functional leg power in a representative cohort of British men and women: associations with physical activity, health status, and socioeconomic conditions. *The Journals of Gerontology Series A: Biological Sciences and Medical Sciences.* 2005;60(2):224-231. doi:10.1093/gerona/60.2.224.
6. Miller MR, Hankinson J, Brusasco V, et al. Standardisation of spirometry. *European respiratory journal.* 2005;26(2):319-338. doi:10.1183/09031936.05.00034805.
7. Cai Y, Shaheen SO, Hardy R, Kuh D, Hansell AL. Birth weight, early childhood growth and lung function in middle to early old age: 1946 British birth cohort. *Thorax.* 2015;71(10):thoraxjnl-2014-206457. doi:10.1136/thoraxjnl-2014-206457.
8. Fuller E, Power C, Shepherd P, Strachan D. *Technical report on the National Child Development Study biomedical survey 2002-2004.* 2006.
9. Richards M, Hardy R, Wadsworth ME. Long-term effects of breast-feeding in a national birth cohort: educational attainment and midlife cognitive function. *Public health nutrition.* 2002;5(5):631-635. doi:10.1079/PHN2002338.
10. Brown M, Elliott J, Hancock M, Shepherd P, Dodgeon B. *National Child Development Study: 2008-2009 Follow-Up: User guide to the data* 2012.
